# P53 and BCL-2 family proteins PUMA and NOXA define competitive fitness in pluripotent cell competition

**DOI:** 10.1371/journal.pgen.1011193

**Published:** 2024-03-15

**Authors:** Jose A. Valverde-Lopez, Lin Li-Bao, Rocío Sierra, Elisa Santos, Giovanna Giovinazzo, Covadonga Díaz-Díaz, Miguel Torres

**Affiliations:** 1 Cardiovascular Regeneration Program, Centro Nacional de Investigaciones Cardiovasculares (CNIC), Madrid, Spain; 2 Centro de Investigación Biomédica en Red de Enfermedades Cardiovasculares (CIBERCV), Madrid, Spain; 3 Pluripotent Cell Technology Unit, Centro Nacional de Investigaciones Cardiovasculares, CNIC, Madrid, Spain; Osaka University: Osaka Daigaku, JAPAN

## Abstract

Cell Competition is a process by which neighboring cells compare their fitness. As a result, viable but suboptimal cells are selectively eliminated in the presence of fitter cells. In the early mammalian embryo, epiblast pluripotent cells undergo extensive Cell Competition, which prevents suboptimal cells from contributing to the newly forming organism. While competitive ability is regulated by MYC in the epiblast, the mechanisms that contribute to competitive fitness in this context are largely unknown. Here, we report that P53 and its pro-apoptotic targets PUMA and NOXA regulate apoptosis susceptibility and competitive fitness in pluripotent cells. PUMA is widely expressed specifically in pluripotent cells *in vitro* and *in vivo*. We found that P53 regulates MYC levels in pluripotent cells, which connects these two Cell Competition pathways, however, MYC and PUMA/NOXA levels are independently regulated by P53. We propose a model that integrates a bifurcated P53 pathway regulating both MYC and PUMA/NOXA levels and determines competitive fitness.

## Author summary

Cells compete with neighbors for survival in several situations *in vitro* and *in vivo*. In mammalian embryos, this phenomenon takes place among pluripotent cells, which are those able to form all the tissues of a new organism. Cell Competition in this context is thought to function as a quality check for the elimination of potentially damaged or dangerous cells (losers) whenever “fitter” cells (winners) are present. Here, we explored the molecular features of winner and loser pluripotent cells and uncovered a pathway that drives the ability of cells to compete. This pathway is governed by p53, a known factor that responds to stress and modulates cell death in several contexts, including cancer. Downstream this pathway, we found that p53 controls Cell Competition by regulating two independent branches; one involving the regulation of MYC, an oncogene previously known to regulate Competition in pluripotent cells, and a second one involving two mitochondrial factors known to regulate cell death and metabolism. Our work uncovers new mechanisms of pluripotent Cell Competition and show that the p53 cellular stress response pathway is involved in this developmental process.

## Introduction

Cell Competition (CC) is a process based on the interaction of neighboring cells of the same type. By this mechanism, less fit cells, known as “*loser cells”*, are non-autonomously eliminated upon confrontation with fitter cells called “*winners*”. Cell competition selectively detects and eliminates viable but suboptimal, mis-specified or mis-placed cells, being envisioned as a conserved and extended quality control system in metazoans. From embryonic development to the adult, Cell Competition functions to ensure the proper performance of tissues and organs. In addition, it plays an important role in aging, tissue regeneration and cancer [1–3].

Cell Competition is particularly active in pluripotent cells of the early mammalian embryo [4–8]. Pluripotency is defined as the capacity of embryonic cells to self-renew and generate all embryonic lineages. Although pluripotent cells maintain a core pluripotency TF regulatory network, pluripotency is not a single status, but a sequence of dynamic stages in which cells change their gene expression, epigenetic landscape and metabolic profile in a continuous manner during development [9,10]. In the mouse embryo, pluripotency extends from E3.5 to E6.5-E7.5 (S1 Fig). The early mouse blastocyst contains two cell populations, the undifferentiated inner cell mass (ICM), composed of pluripotent cells, and the trophectoderm, composed of differentiating cells. Before implantation, the ICM segregates into the primitive endoderm and the epiblast (S1 Fig). At this stage the epiblast exhibits a "naïve" pluripotent status characterized by a broadly hypomethylated "open" chromatin configuration. After implantation, epiblast cells progress through a stage of "formative pluripotency" (S1 Fig), during which, cells acquire extensive methylation marks and establish X-chromosome inactivation [11]. By E6.5, epiblast cells enter the "primed" pluripotent status in which they are poised for their differentiation during gastrulation into the three germ layers; ectoderm, mesoderm and endoderm (S1 Fig) [12,13].

Starting at epiblast specification, pluripotent cells are susceptible to undergo apoptosis, with a wave of cell death that peaks at the pre-gastrulation epiblast in a programmed and systematic manner. At this stage, cells become hypersensitive to apoptotic stimuli [14–18]. This apoptosis wave ends with gastrulation, which also coincides with termination of pluripotency. At least in part, this cell death wave results from endogenous Cell Competition that eliminates prematurely differentiating, suboptimal or potentially harmful cells in presence of fitter cells. This process optimizes the cell pool that will give rise to the new individual [4–8,19]. In this endogenous CC model, winner cells show high expression of MYC and low expression of P53 transcription factors. Cells with low MYC levels are eliminated by the presence of cells with higher levels, as a mechanism for selecting metabolically competent cells and removing cells that differentiate prematurely, which safeguards pluripotency [4–6]. Similar observations apply to the *in vitro* counterparts of epiblast cells as they transit from naive to primed pluripotency and differentiation (S1 Fig). Although different pathways have been reported to regulate fitness in pluripotent CC [4,5,20–23], many aspects of the molecular mechanisms involved remain unknown. In particular, how competitive fitness is encoded in pluripotent cells is not understood.

Identifying pre-existing features of prospective loser cells contributing to their loser status would provide insight into the early steps of CC and fitness comparison. Here, we have explored different factors and pathways regulating cell fitness and underlying the execution of loser cell death during pluripotent CC. As a result, we have identified several candidates of the P53 pathway and investigated their role in pluripotent CC. We identified P53 pathway upregulation in loser ES cells and show its essential role in regulating Myc levels and acquisition of the loser phenotype. We report that P53 and its pro-apoptotic BCL2-family mitochondrial targets PUMA and NOXA regulate apoptosis susceptibility in ESCs in a Myc-independent manner. The function and expression of this pathway in pluripotent cells is not restricted to dying cells, but present in all the cell population, where its inhibition is enough to promote the winner phenotype. P53 regulation of competitive fitness depends on the pluripotency status, with the pathway being activated as the cells progress towards differentiation, which increases apoptotic hypersensitivity. In contrast, the ability of the pathway to induce CC and apoptosis is suppressed in naive pluripotency conditions. Based on these results, we propose a model that integrates the P53 and MYC pathways in the definition of the loser cell fitness “status” during pluripotency progression in mammalian embryonic cells.

## Results

### The P53 pathway is upregulated in MYC-low embryonic stem cells

**Fig 1:**
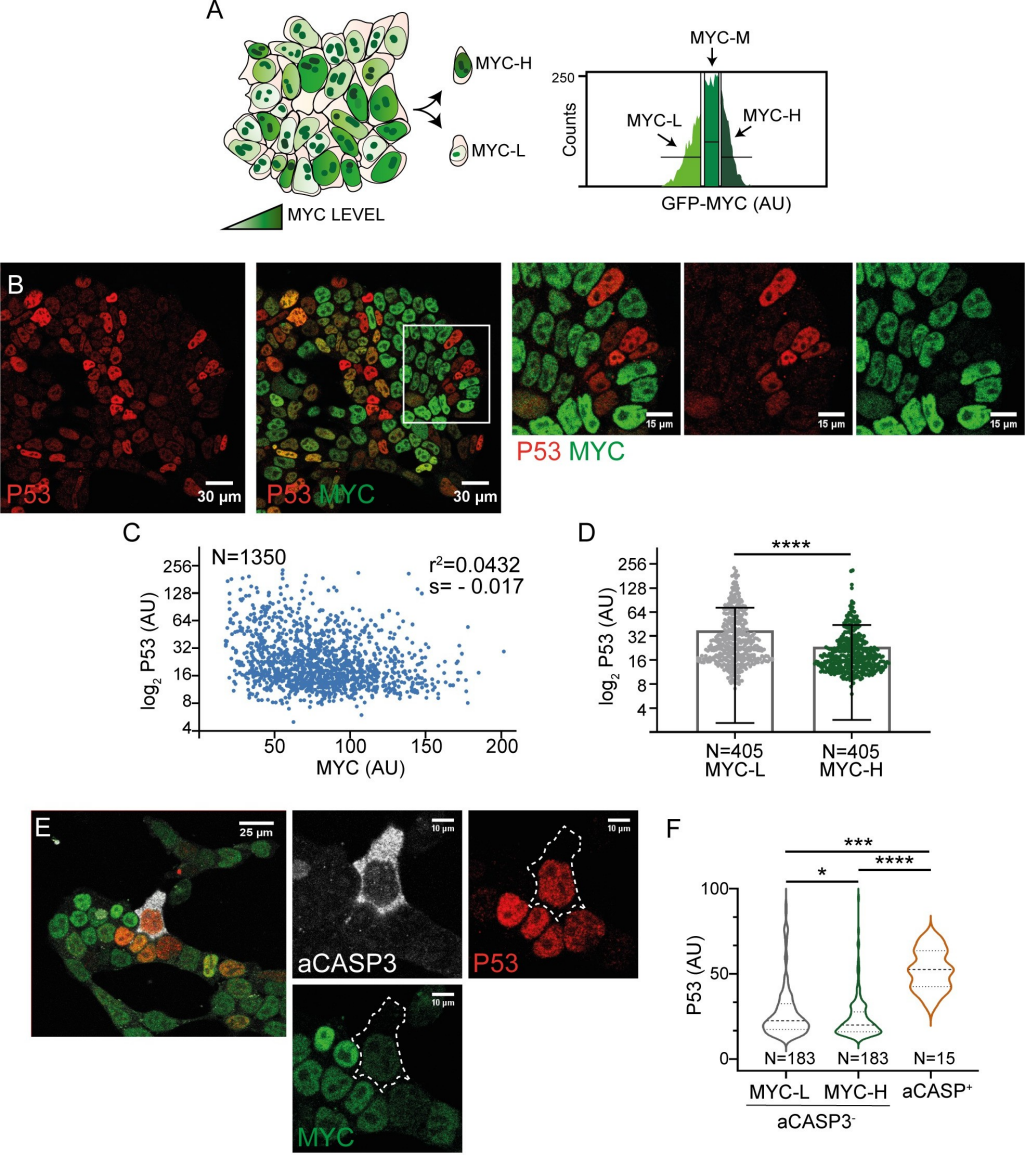
The P53 pathway is upregulated in MYC-low ES cells. **A.** Schematic representation of the GFP-MYC ES cell line (left). Histogram showing the segmentation of GFP-MYC ES cells into three populations (MYC-low, -medium and -high), which were sorted by FACS (right). **B.** Confocal images showing the expression of P53 and MYC in ES cells and magnification (right). **C.** Quantification of P53 and MYC levels, p<0.0001. **D.** P53 levels in MYC-low and MYC-high populations. **E.** Confocal images showing active CASP3, P53 and MYC immunostaining and magnification (right). **F.** Quantification of P53 in active CASP3 positive cells and in MYC-low and MYC-high aCASP3 negative cells.

To identify pathways involved in the regulation of cellular fitness and the execution of loser cells elimination, we compared the transcriptional profile of low- and high-MYC expressing ES cells. Except when indicated otherwise, ES cells were cultured in the “FBS+LIF” medium described in Materials and Methods. In a previous work, we performed transcriptomic studies comparing low-, medium- and high-MYC expressing cells [6]. We took advantage of a GFP-MYC reporting cell line, in which GFP levels reliably report MYC expression. GFP-MYC ES cells were sorted by FACS according to GFP expression levels and their transcriptome was sequenced (Fig 1A). This procedure allowed us to study candidate genes in the MYC-low cell population potentially involved in their low competitive ability or responsible for their elimination. Using gene-set enrichment analysis to reanalyze the data in [6], we identified P53 as the most enriched pathway in MYC-low cells (S2A Fig). To validate this correlation between P53 and MYC-low cells, we performed an immunostaining in ES cells. P53 exhibited a heterogeneous nuclear pattern in ES cells (Figs 1B and S2B) and showed a strong increase upon treatment with etoposide (widely P53 activator through DNA damage generation) as a positive control (S2B Fig). Per-cell quantification of P53 and MYC expression confirmed an inverse correlation between the two proteins (Fig 1B–1D). Then, we checked the apoptotic role of P53 in ES cells by using an anti-active CASP3 antibody. To avoid problems of apoptosis-associated autofluorescence, only cells that maintain an integral cellular morphology (early apoptotic cells) were considered for this quantification (Fig 1E). Activated CASP3-positive cells displayed higher levels of P53, indicating a correlation between P53 and apoptosis (Fig 1F). Additionally, when considering CASP3 negative cells only, P53 expression was still higher in MYC-low cells than in MYC-high cells (Fig 1E and 1F) [6]. These observations suggest a role for P53 pathway in the execution of loser cell death but also indicate that P53 could exert a role in defining fitness and the loser “status” in the general cell population. Therefore, we focused on selecting candidate genes of the P53 pathway involved in apoptosis/cell stress, upregulated in MYC-low cells.

**Fig 2:**
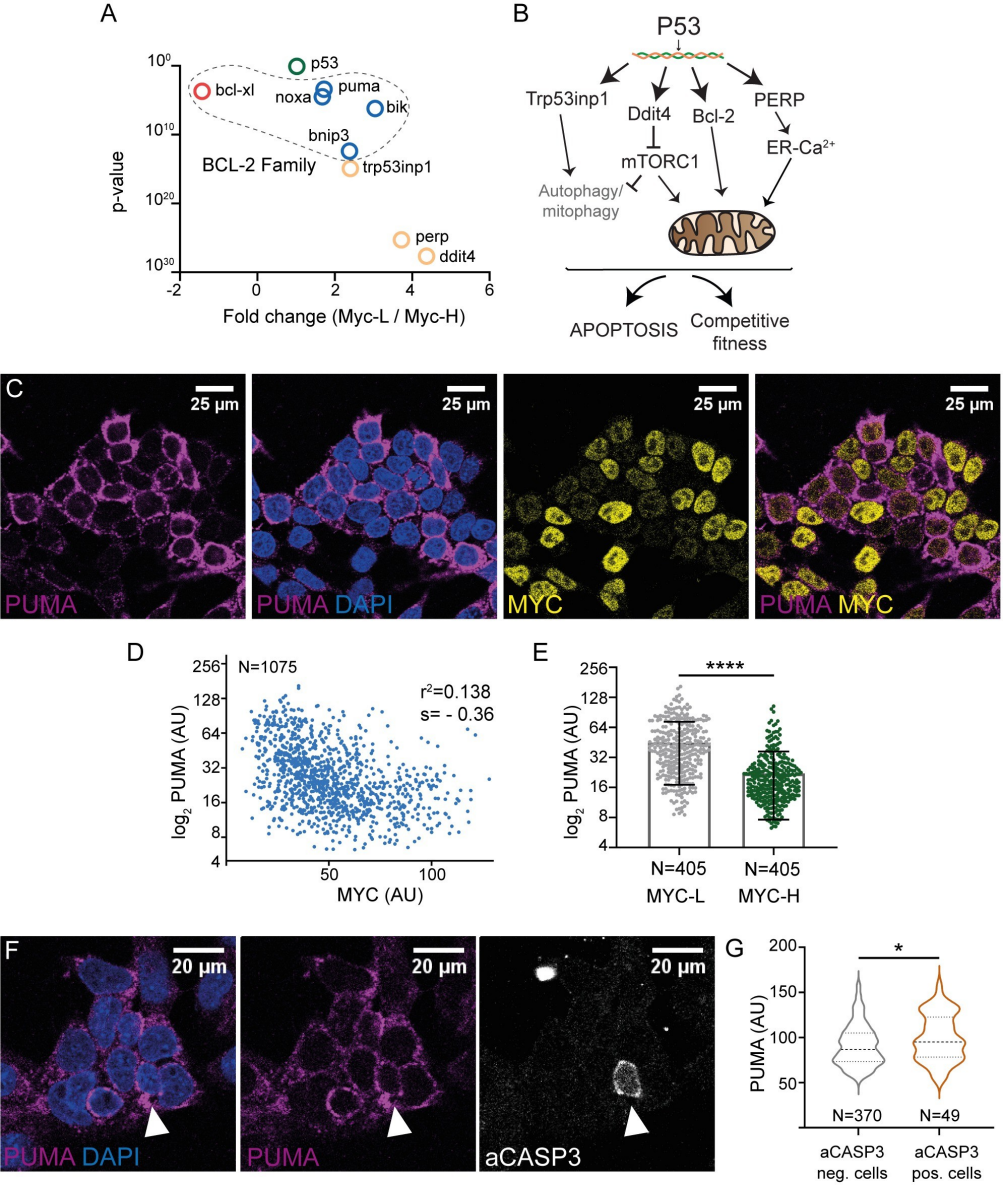
Candidate genes upregulated in MYC-low cells. PUMA levels inversely correlate with MYC levels. **A.** Dot plot showing the fold change and p value of candidate genes. **B.** Schematic representation of different candidate genes related to the P53 pathway and involved in apoptosis/stress, upregulated specifically in MYC-low cells. **C.** Confocal images showing PUMA and MYC expression in ESCs. **D, E**. Quantification of PUMA and MYC expression, p<0.0001 and expression of PUMA in MYC-L and MYC-H cells. **F.** Confocal images of aCASP3 and PUMA immunostaining. **G.** Quantification of PUMA levels in aCASP3 positive and negative cells.

Using gene ontology (GO) terms related to P53 pathway and apoptosis, we were able to select from the transcriptomic analysis those genes involved in the P53 pathway and apoptosis (GO terms are described in Materials and Methods and the list of candidate genes can be found in Tables A-D in S3 Table). We identified a variety of genes involved in stress response and apoptosis, such as *trp53inp1*, *ddit4* or *perp* and members of the BCL-2 family (Figs 2A, 2B and S2C). The higher expression of these candidate genes in MYC-low cells was analyzed by qPCR, which confirmed the RNAseq results (S2D Fig).

*Trp53inp1* is a stress-induced protein that induces autophagy and mitophagy [24]. *Ddit4* is upregulated upon stress and affects mitochondrial biogenesis and metabolism. It functions as a strong inhibitor of mTORC1, which induces autophagy [25]. *Perp* encodes a plasma membrane protein which can interact with the Ca^2+^ pump (SERCA2B) in the endoplasmic reticulum, inducing apoptosis [26].

BCL-2 (B cell lymphoma-2) proteins constitute important regulators of apoptosis (Fig 2B). Structurally, these proteins possess a conserved BH1-4 domains (BCL-2 homology 1–4), critical for their function. They are classified into multi-BH factors, including anti-apoptotic (BLC-2, BCL-XL, MCL-1 and others) and pro-apoptotic factors (BAX, BAK, BOK) or BH3-only pro-apoptotic factors (BIM, BAD, tBID, NOXA, PUMA and others) (S3A Fig). Upon apoptotic stimuli, multi-BH pro-apoptotic proteins BAX, BAK and BOK can oligomerize and generate pores in the mitochondrial outer membrane (MOM) allowing pro-apoptotic factors to release and trigger apoptosis. This oligomerization is tightly controlled by the balance between anti- and pro- apoptotic BCL2 proteins [27] (S3B Fig).

From this family, we analyzed the expression of PUMA (*bbc3*), one of the most important apoptotic factors downstream P53 [28]. PUMA was detected in almost all ESCs by immunostaining, exhibiting a cytosolic pattern with variable levels of expression (Fig 2C). Per-cell quantification of PUMA and MYC levels revealed an inverse correlation, which was confirmed by immunoblot (Figs 2D, 2E and S3C). Then, we performed aCASP3 staining, which showed that apoptotic cells expressed moderately higher PUMA levels with respect to the general cell population (Fig 2F and 2G). Using Reverse-transcription PCR, we found that *puma* upregulation in MYC-low cells corresponds to the main isoform, isoform 1 (S3D Fig).

The fact that PUMA is heterogeneously expressed in almost all cells and that apoptotic cells show only moderately higher PUMA levels suggests that PUMA roles extend beyond the mere execution of apoptosis. Considering the inverse correlation between PUMA and MYC, and that MYC is a well-described fitness regulator, we hypothesized that PUMA could play a role in regulating competitive fitness.

### P53-PUMA and MYC regulation in pluripotent cells

**Fig 3:**
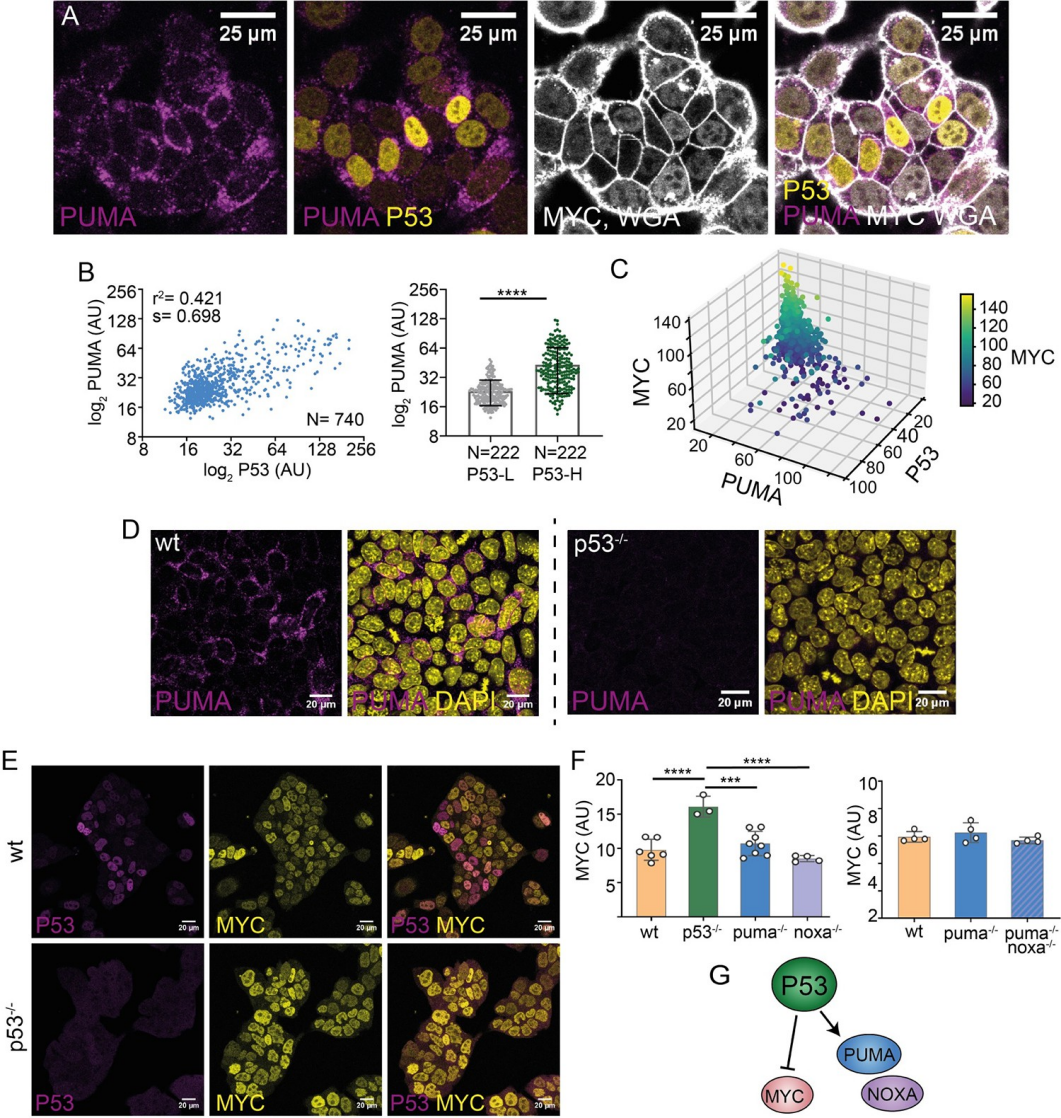
P53 regulation of PUMA and MYC in ES cells. **A.** Confocal images showing P53, PUMA and MYC expression. **B.** Quantification of P53 and PUMA expression. **C.** Quantification of P53, PUMA and MYC levels, N = 384, PUMA-MYC (r^2^ = -0.506, p = 2.09x10^-26^), P53-MYC (r^2^ = -0.457, p = 2.09x10^-21^), P53-PUMA (r^2^ = 0.669, p = 3.50x10^-51^). Graph generated with Python. Heatmap corresponds to MYC expression. **D.** Confocal captures showing PUMA levels in *WT* and *p53*^*-/-*^ cells. **E.** Confocal images of P53 and MYC levels in *WT* and *p53*^*-/-*^ cells. **F.** MYC levels in *WT*, *p53*^*-/-*^, *puma*^*-/-*^, *noxa*^*-/-*^ (left) and (right) *WT*, *puma*^*-/-*^ and the double knockout *puma*^*-/-*^and *noxa*^*-/-*^. This experiment was analyzed by FACS, where each dots represent a different ES cell line. At least ten thousand events were analyzed for each ESC clone. **G.** Schematic representation of P53 regulating the expression of PUMA and NOXA while exerting an inhibitory effect over MYC expression.

First, we studied the regulatory interactions between P53 and PUMA. To do so, we performed a triple immunostaining against P53, PUMA and MYC proteins and found a positive correlation between P53 and PUMA levels in ESCs (Fig 3A and 3B), and an inverse correlation of both of them with MYC levels (Fig 3A and 3C). In similarity to the observations with p53, etoposide efficiently upregulated PUMA levels in ESCs (S4A and S4B Fig). We then generated a *p53* knockout ES cell line and checked PUMA expression. Notably, in the absence of P53, we observed no detectable PUMA signal in ES cells (Fig 3D), indicating that P53 is absolutely required for PUMA expression in this context.

Next, we wanted to explore the regulatory interactions between MYC and the P53-PUMA pathway. First, we analyzed the levels of P53 and PUMA using a *myc* knockout cell line and we found that MYC deletion did not increase P53 or PUMA expression, but rather we observed a slight non-significant downregulation (S4C and S4D Fig). Subsequently, we examined the expression of P53 and PUMA when MYC is overexpressed, taking advantage of the mouse ES cells carrying the *iMOS*^*MYC*^ allele [4]. Upon tamoxifen exposure, mutually exclusive populations of EYFP-positive cells overexpressing MYC and of wild type (WT) ECFP-positive cells are induced in a random mosaic manner at reproducible proportions (S4E Fig). We found that MYC-overexpressing and WT cells showed similar levels of P53 and PUMA expression (S4E Fig). These results show that MYC does not regulate P53-PUMA expression in ESCs.

Then, we analyzed whether, alternatively, P53 regulates MYC. We found that in P53-KO ESCs, MYC was upregulated (Fig 3E and 3F). Additionally, activation of P53 using Nutlin3 (an extensively characterized P53 activator) led to MYC downregulation (S5A–S5C Fig). In similarity to etoposide, Nutlin3 also led to the upregulation of PUMA expression.

Next, we studied whether PUMA and a second BH3-only p53 target; NOXA (*pmaip1*), regulate MYC expression in ESCs. We generated ESC lines mutant for either *puma*, *pmaip1* (*noxa* from here on) or both, and studied MYC expression. In the absence of either PUMA or NOXA, or both, we did not find changes in MYC expression (Fig 3F). These results indicate that P53 regulates positively PUMA/NOXA and negatively MYC as independent pathways (Fig 3G).

**Fig 4:**
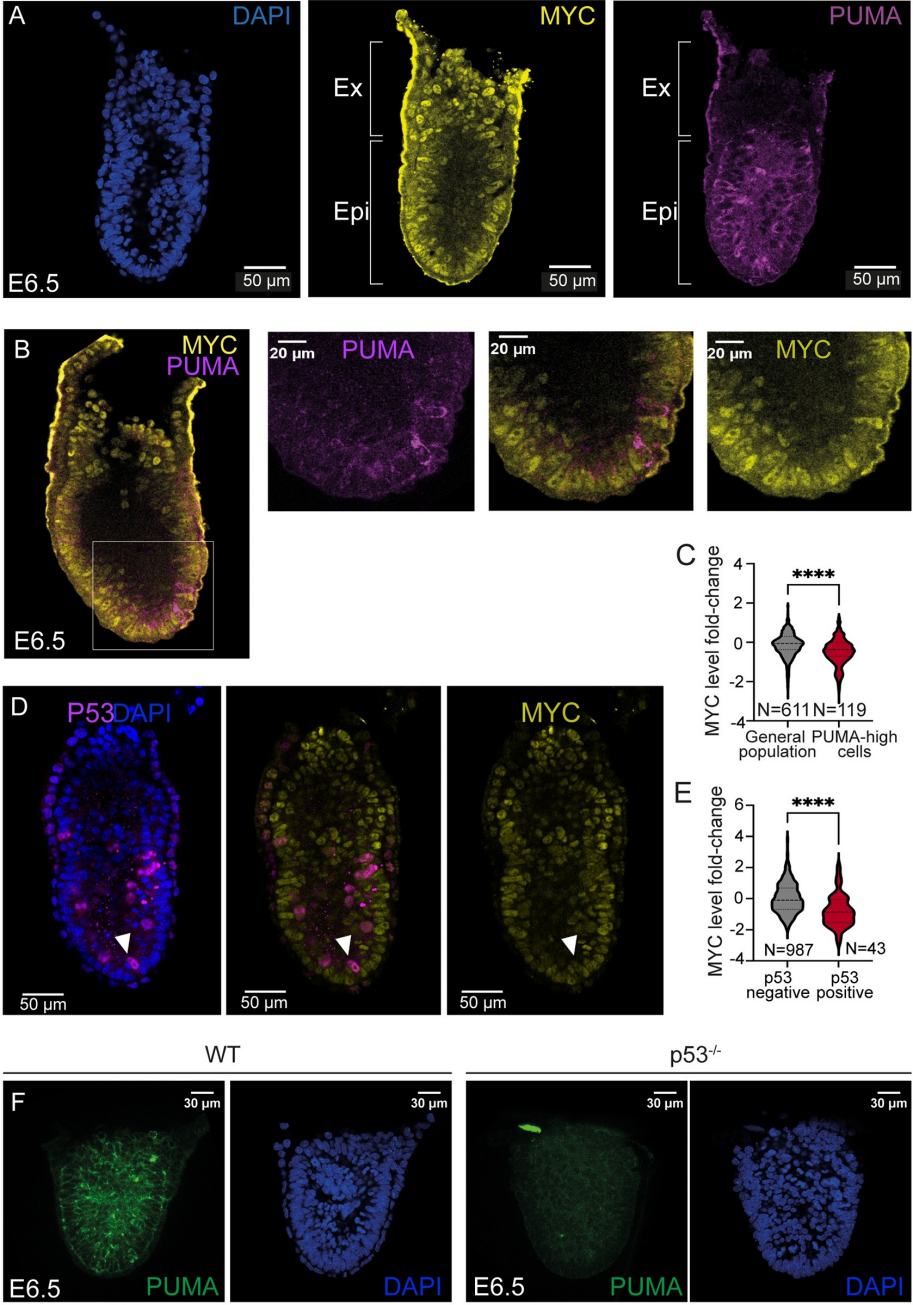
P53, PUMA and MYC expression and regulation in the early mouse embryo. **A, B.** Confocal images showing PUMA and MYC expression in E6.5 mouse embryos (Ex, extraembryonic ectoderm; Epi, epiblast). **C**. Quantification of normalized MYC levels in the general cell population and high PUMA-expressing cells. **D** Confocal detection of P53 and MYC expression in an E6.5 mouse embryo. **E.** Normalized MYC-level distributions in P53 positive and P53 negative cells of the mouse E6.5 epiblast. (N = 4 embryos). **F**. Confocal images showing PUMA expression in *WT and p53*^*-/-*^ E6.5 embryos. (N = 16 embryos).

We then explored P53-PUMA and MYC correlation *in vivo* in the early mouse embryo. E6.5 mouse embryos were first studied, given that MYC-driven Cell Competition and other CC models have been described at this developmental stage [4–6,20,21]. In agreement with the observations in ES cells, E6.5 epiblast cells expressed heterogeneous levels of PUMA (Fig 4A). In contrast to the epiblast, the extraembryonic ectoderm (Ex) did not show detectable PUMA expression, while MYC is strongly expressed there (Fig 4A), indicating that co-expression only takes place in embryonic pluripotent cells. We found that epiblast cells with high PUMA levels exhibit lower MYC levels than the general cell population (Fig 4B and 4C). Next, we explored the correlation between P53 and MYC expression levels in the E6.5 epiblast and again found a negative correlation between the two transcription factors (Fig 4D and 4E).

To determine the functional role of P53 in regulating MYC expression *in vivo*, we studied *p53* KO embryos and determined MYC levels (S6A Fig). Contrary to the results obtained *in vitro* (Fig 3E), we did not observe any change of MYC levels in E6.5 *p53* KO embryos (S6A Fig). In contrast, we found that *p53* KO embryos completely lack PUMA expression at E6.5 (Fig 4F), which reproduces the previous results *in vitro* (Fig 3D).

MYC-mediated CC interactions have also been described in preimplantation embryos [22]; therefore, we also studied embryos at E3.5. In embryos at this stage, P53 showed a nuclear pattern analogous to that in ES cells (S6B Fig) and we found a positive correlation between P53 and PUMA expression levels (S6B and S6C Fig). In contrast, no correlation was found between P53 and MYC (S6D and S6E Fig). We next studied whether P53 regulates MYC in embryos at this stage. In similarity to the observations with E6.5 embryos (S6A Fig), we did not find any changes of MYC expression levels in E3.5 *p53* KO embryos (S6F Fig). These results show that in the early embryo, MYC regulation by P53 is not as simple as in cultured ESCs and additional factors may add complexity to the regulatory network.

### P53-PUMA and MYC are regulated accordingly to the pluripotency status

We previously described that MYC is regulated by the pluripotency status [6], thus, we explored the relationship between Pluripotency status and the P53-PUMA pathway. Different pluripotent states can be recapitulated *in vitro* (S1 Fig). The use of two differentiation inhibitors, PD03 and CHIRON (known as 2i), maintains cells in a pluripotent state that resembles the naive status [29,30]. Cells cultured in Fetal Bovine Serum (FBS) or “KO Serum Replacement” (SR) plus LIF retain stemness and self-renewal properties, but also receive signals that promote progression into formative pluripotency. This results in a culture with heterogeneous pluripotent states (S1 Fig) [6,31]. Primed cells can be obtained from the epiblast cells of the post-implantation mouse embryo and maintained in a medium containing Activin A and FGF (S1 Fig) [32,33]. Removal of LIF from the SR+LIF or FBS+LIF results in transition through the formative and primed states followed by differentiation (S1 Fig).

**Fig 5:**
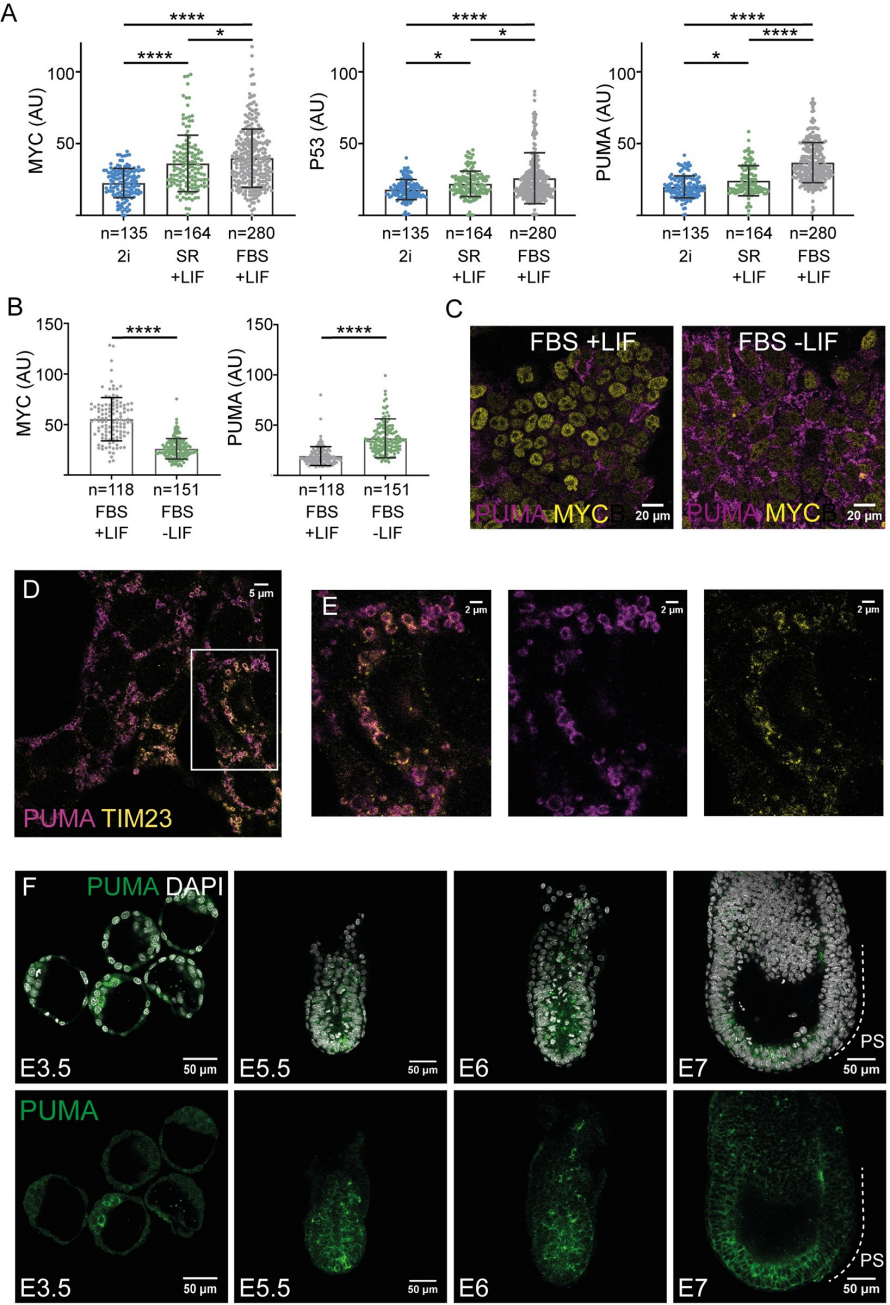
The pluripotency status regulates P53 and PUMA expression. **A.** Quantification of MYC, P53 and PUMA in 2i, SR + LIF and FBS + LIF conditions. For the different conditions: 2i n = 135, SR+LIF n = 164, FBS+LIF n = 280 **B.** Quantification of MYC and PUMA expression in conventional or differentiating conditions (FBS, 3 days after removing LIF). **C.** Confocal detection of PUMA and MYC expression in FBS+LIF and FBS, 3 days after removing LIF. **D, E** STED-Super-resolution microscopy images showing PUMA and mitochondrial marker TIM23 levels. **F.** Confocal images displaying PUMA expression pattern in mouse early embryos at the indicated stages. PUMA expression pattern has been checked in at least four embryos for each stage.

Here, we analyzed P53, PUMA and MYC expression dynamics in culture conditions that promote different pluripotency states: FBS+LIF+2i (2i), FBS+LIF and SR+LIF. We found that P53, PUMA and MYC expression levels increased as cells transit from naive into mixed pluripotency (Fig 5A). Furthermore, removing LIF for 3 days, led to an increase in PUMA levels, and a decrease in MYC levels (Fig 5B and 5C). Collectively, these results indicated that MYC and PUMA levels increase as cells progress towards primed pluripotency, while they diverge as cells approach differentiation. During transition from naive to formative pluripotency the MYC and the p53 pathways thus correlate at the population level; however, as shown above (Figs 2C–2E and 3), at the individual cell level, they show a negative correlation.

We then explored the subcellular localization of PUMA in ESCs, taking advantage of super-resolution STED microscopy. We found that PUMA strongly co-localizes with the mitochondrial membranes (Fig 5D and 5E). This observation agrees with previous characterizations of PUMA mitochondrial localization [28] and suggests that its roles in pluripotent cells relate to mitochondrial functions.

Next, we extended the study of the expression pattern of PUMA during early mouse embryo development to all pluripotency stages. At E3.5, PUMA levels were low or not detected except from some ICM cells showing higher expression levels. Subsequently, from E5 to E6.5, PUMA is heterogeneously expressed in the epiblast, which recapitulates the observations in ES cell cultures. Eventually, when gastrulation begins and pluripotency ends, PUMA levels strongly decreased in the gastrulating cells of the primitive streak (Fig 5F). High expression of PUMA at heterogeneous levels therefore seems to be related to pluripotency *in vitro* and *in vivo*, while definitive cell differentiation in the primitive streak terminates PUMA expression. This expression dynamic is parallel to that of MYC at the level of the whole cell population, given that MYC shows a heterogeneous expression pattern until gastrulation, when its expression first disappears from the primitive streak [4,6]. Nonetheless, in similarity to the *in vitro* conditions, the expression levels of PUMA and MYC *in vivo* are anti-correlated at the single-cell level before primitive streak formation (Fig 4B and 4C). This anticorrelation, however, was not detected in the naive epiblast of the 3.5 embryo between P53 and MYC (S6D and S6E Fig), indicating that it is a characteristic of the post-implantation epiblast during formative pluripotency.

### Functional characterization of P53 and the BH3-only proteins PUMA and NOXA

**Fig 6:**
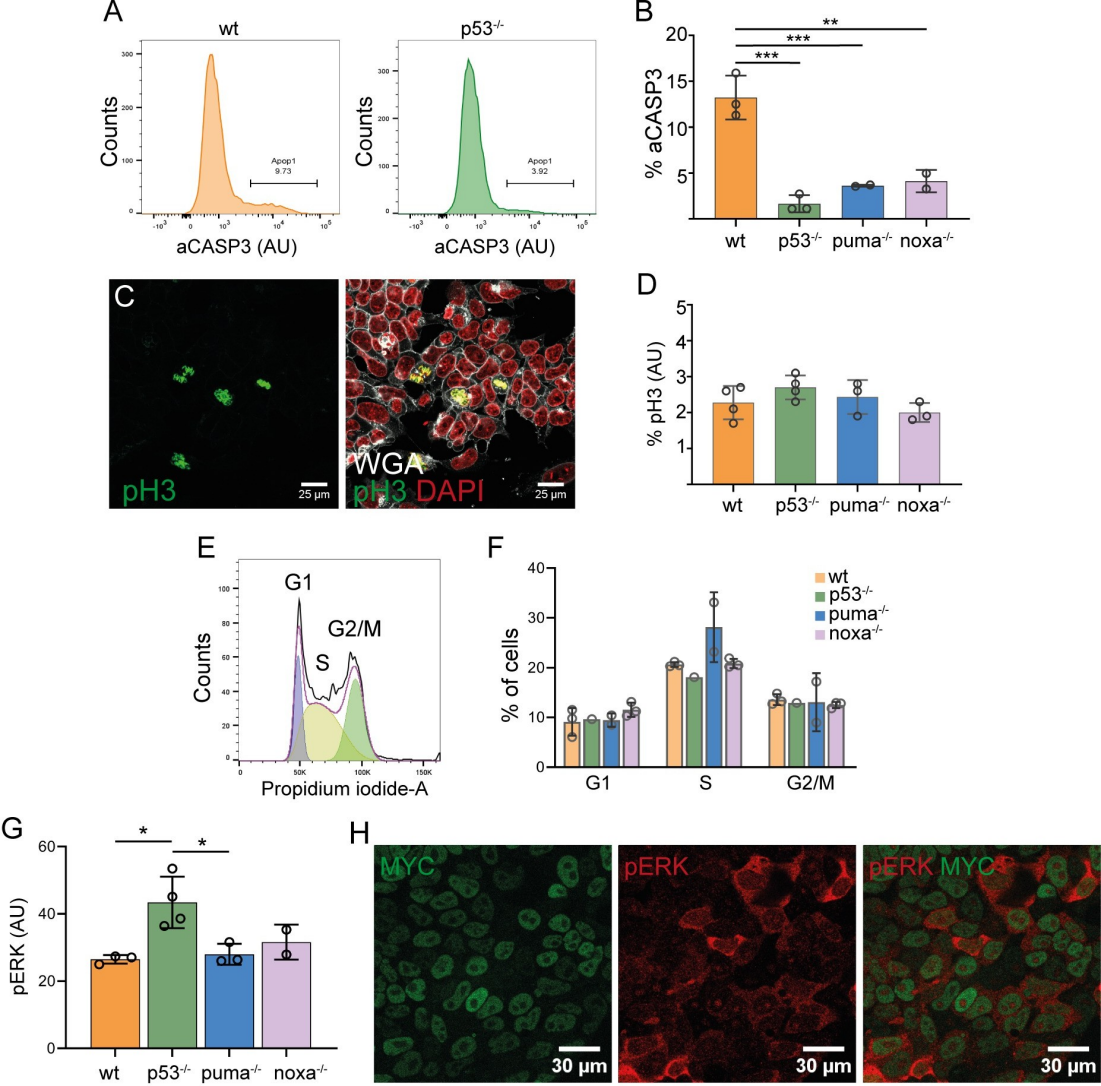
Role of P53 and BH3-only protein PUMA and NOXA in ESC apoptosis, proliferation and cell cycle. **A.** Histograms showing aCASP3 staining in *WT* and *p53*^***-/-***^ cells. **B.** Quantification of the percentage of aCASP3 positive cells. **C.** Confocal images showing phH3 positive cells and quantification (**D**). **E.** Histogram showing cell cycle and quantification (**F**). **G.** Bar graph showing p-ERK levels and confocal captures of p-ERK and MYC expression in ESCs (**H**). “aCasp3” refers to the active or cleaved Caspase3 protein. “pH3” refers to the phosphorylated Histone 3 protein.

After analyzing the P53-PUMA and MYC regulation, we studied their function in ES cells. P53 is well known for inducing cell cycle arrest and apoptosis in response to DNA damage and more recently it has been also related to other functions such as autophagy, metabolism or differentiation [34]. On its side, BH3-only proteins are mostly related to apoptosis, although other functions such as metabolic regulation have been recently described [35,36]. In ES cells, the role of P53 in apoptosis and cell cycle arrest is not clear and recent works suggest that P53 functions vary as ESCs progress through the pluripotent states [37–39]. Regarding PUMA and NOXA, their role in Pluripotent Stem Cells is for the most part unknown. To test the role of P53, PUMA and NOXA in apoptosis, cell cycle arrest and differentiation, we used the knockout ESC lines. By performing immunostaining against aCASP3, we found that in the absence of either P53, PUMA or NOXA there was a decrease in apoptosis (Fig 6A and 6B). Similar results were obtained by using a fluorogenic substrate of CASP3/7 (FLICA) (S7A Fig).

We then evaluated the proliferation and cell cycle by analyzing phospho-Histone 3 (pH3) immunostaining and propidium iodide staining respectively. The absence of either P53, PUMA or NOXA did not lead to significant changes in proliferation (Figs 6C, 6D and S7B) or in the proportion of cells at different phases of the cell cycle (Fig 6E and 6F).

We finally explored how the elimination of P53 and its targets affect ESC differentiation status. We found that p-ERK, a marker of primed ESCs [40], is expressed at higher levels in *p53* KO cells than in control ESCs (Fig 6G). In the same test, as previously described [6], Myc and p-ERK show an inverse correlation (Fig 6H). This suggests a role of P53 in ESC transition from pluripotency to differentiation. In contrast, *puma* or *noxa* elimination does not modify p-ERK expression (Fig 6G).

### P53 and BH3-only proteins PUMA and NOXA regulate cell competitive fitness

Here, we have identified P53 and its targets PUMA and NOXA, as upregulated in MYC-low cells during Cell Competition and we found a role for these factors in ESC susceptibility to apoptosis. The fact that P53 and PUMA are widely expressed in pluripotent cells and that they exhibit a strong inverse correlation with MYC levels suggests they can contribute to define competitive fitness. As opposed to a pro-apoptotic factor simply involved in the culling of loser cells, a fitness component is expected to change the dynamics of cell competition in a cell non-autonomous manner. To study this aspect, we studied how the elimination of these factors affects the viability of neighboring cells in competition assays. We performed these experiments in cells starting differentiation, as at this stage the competitive interactions are enhanced [5,7,20,41].

**Fig 7:**
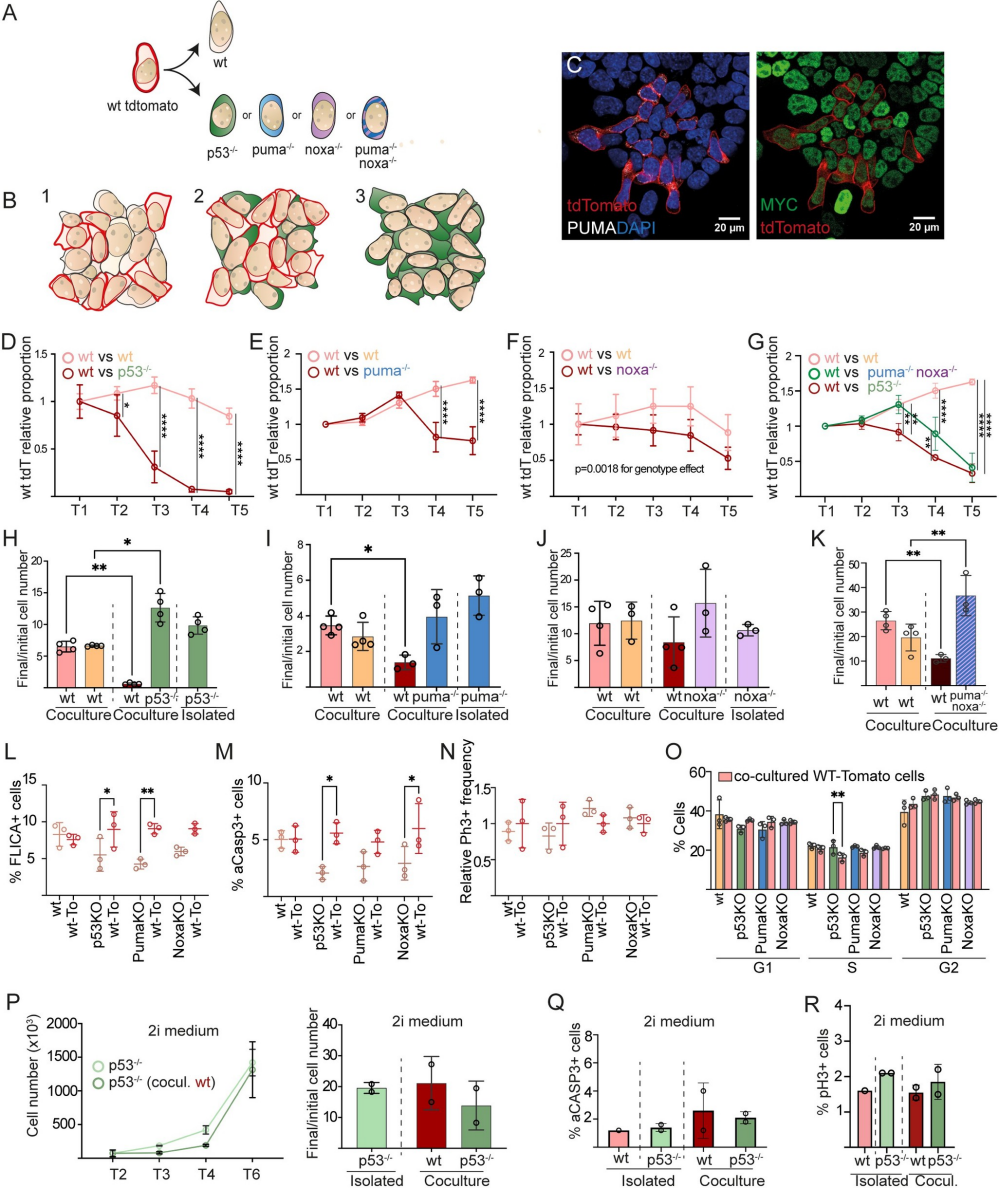
P53, PUMA and NOXA regulate competitive fitness. **A.** Schematic representation of the experimental design. *Tomato*-*WT* cells were confronted either with knockout cells (represented in different colors) or non-fluorescent *WT* cells as a control. **B.** Schematic representation showing the three types of cell culture we monitor in this assay. (1) *Tomato*-*WT* in co-culture with non-fluorescent *WT* cells, (2) *Tomato*-*WT* in co-culture with the different knockout cell lines. Here *p53*^*-/-*^ cells, used as an example of knockout cell line, are represented in green. (3) Homotypic culture of the different knockout cell lines. **C.** Confocal image showing *Tomato*-*WT* and *puma* knockout cells. **D-G**, Evolution of *Tomato*-*WT* cell number in co-culture with either *WT* cells or *p53*^*-/-*^ cells **(D)**, *puma*^*-/-*^ **(E)**, noxa^*-/-*^ **(F)** or double knockout *puma*^*-/-*^; noxa^*-/-*^ cells in parallel to *p53*^*-/-*^ cells **(G)**. Experiments in E and G were done simultaneously but shown separately for better visualization. **H-K**, Bar plots representing the ratio between the final and initial cell number of *WT* and *p53*^*-/-*^, *puma*^*-/-*^, *noxa*^*-/-*^ or the double knockout *puma*^*-/-*^, noxa^*-/-*^ cells in isolated or in co-culture conditions. Each dot represents a different clone. **L**, **M**, apoptosis rate estimation during competition by measurement in cytometry of Caspase 3/7 activity using FLICA (**L**) and of immunodetection of activated Caspase 3 (**M**). **N**, **O**, measurement of cell cycle activity during competition by PH3+ cell detection in confocal analysis (**N**) and by cell cycle phase detection in cytometry (**O**). **P** (left), Evolution of the *p53*^*-/-*^ cell population isolated or in co-culture with *WT* cells. **P** (right), final/initial cell number ratio of *p53*^*-/-*^ cells and *WT* cells isolated and in co-culture. **Q.** Percentage of aCASP3 positive *WT* and *p53*^*-/-*^ cells in the indicated conditions **R.** Percentage of phH3 positive *WT* and *p53*^*-/-*^ in the indicated conditions.

To do so, we confronted *tomato-*expressing *WT* cells with either *p53*, *puma* or *noxa* knockout cells and with non-fluorescent *WT* cells as a control. During CC assays, we compared the evolution of *tomato*-*WT* cells in co-culture with *WT* or the different knockout cell lines (Fig 7A–7C). Additionally, we analyzed the evolution of each knockout cell line growing in homotypic conditions (Fig 7B). *Tomato-WT* cells were eliminated when co-cultured with *p53*^*-/-*^ cells but not when they were co-cultured with other *WT* cells (Fig 7D and 7H). The population of *tomato*-*WT* cells was also reduced when confronted with *puma*^*-/-*^ cells (Fig 7E and 7I). Same experiments with *noxa*^*-/-*^ cells resulted in reduced growth of the confronted *tomato*-*WT* cells to a lesser extent (Fig 7F and 7J). Notably, double knockout *puma*^*-/-*^*; noxa*^*-/-*^ cells produced a stronger reduction in the population of *tomato*-*WT* cells than single *noxa*^*-/-*^ or *puma*^*-/-*^ cells (Fig 7G and 7K), however, the evolution of the cell populations still denoted a stronger effect of eliminating *p53* (Fig 7G). These results indicate that P53 and BH3-only proteins PUMA and NOXA regulate competitive fitness in ES cells in such a way that lower levels of P53, PUMA or NOXA results in higher fitness.

To study whether the observed competition results from differences in cell proliferation or cell death, we measured Caspase 3/7 activity in cytometry using FLICA or detected cells expressing activated Caspase 3 using a specific antibody. We observed in *WT* cells higher rates of Caspase activation than in co-cultured cells mutant for either *p53*, *noxa* or *puma* (Fig 7L and 7M). In contrast to these results, we did not observe differences in the mitotic rate (measured by PH3+ detection) between the confronted populations for any condition (Fig 7N). We also measured the proportion of cells in the different phases of the cell cycle by cytometry and only found a very minor increase in the proportion of cells in S-phase for p53 KO cells (Fig 7O). These results show that the most important factor contributing to CC in this context is cell death and not cell proliferation.

Previous reports showed that CC driven by different pathways does not take place in naive pluripotent cells [5,20,23]. In some studies it was reported that in naive conditions *p53*^*-/-*^ cells behave as losers instead of winners [41]. In agreement with Montero and colleagues [23], we found that in 2i conditions, *p53*^*-/-*^ cells do not show any competitive advantage or disadvantage with respect to WT cells (Fig 7P). Interestingly, in naive conditions, *WT* and *p53*^*-/-*^ ESCs show a similar and small proportion of cells with activated Caspase 3 (Fig 7Q) and showed no differences in mitotic index (Fig 7R).

These results indicate that in naive conditions P53 does not regulate cell death or competitiveness. These observations are in agreement with the low expression of P53 that we have reported in naive ESCs compared to those progressing towards differentiation (Fig 5A) and with the absence of correlation between p53 and Myc levels in the naive epiblast (S6D Fig).

## Discussion

Cell Competition is a mechanism that selects for the fittest cells based on the elimination of viable but suboptimal cells. CC is considered a quality control system extensively conserved in metazoans that may play fundamental roles during development, aging, homeostasis and disease progression [1,42–44]. This mechanism can function as a tumour suppressor mechanism but can also be adopted by tumour cells to promote their own expansion at the expense of neighbouring normal cells [45,46].

In particular, in the early mouse embryo, CC selects the pool of pluripotent cells that eventually will give rise to the new individual. In the early mouse embryo, CC selects cells with higher anabolic activity driven by a YAP-MYC pathway [4] while it removes prematurely differentiating cells [6], cells defective in growth signal detection [5], aneuploid cells [5,8,19] and those cells exhibiting a mitochondrial stress signature [21]. Indeed, cell stress constitutes a pivotal aspect in CC, being described in multiple tissues and CC models [21,47–49].

In an effort to better understand how fitness is regulated and the factors that define the “loser status” in MYC-mediated pluripotent CC, we identified the P53 pathway. P53 is a master regulator and reporter of cell stress and is extensively described in mammalian CC [6,20,23,50–54].

Downstream the P53 pathway, we have identified different candidate genes that can exert a role in the execution of loser cell death and in defining fitness (Fig 2B). We identified candidate factors related to activation of autophagy/mitophagy such as *trp53inp1* or the mTOR inhibitor *ddit4*, Ca^+2^ regulation in endoplasmic reticulum (*perp*) or apoptosis (BH3 proteins) (S8A Fig). We have confirmed that P53 has a role inducing CC and have shown that its BH3-only target PUMA shows widespread expression in pluripotent cells with expression levels inversely correlating with competitive fitness. Together with NOXA, another P53 BH3-only target, PUMA determines pluripotent cell competitive fitness. These results indicate a widespread function of the BH3-only factors PUMA and NOXA beyond the mere execution of apoptosis downstream P53. Nonetheless, *p53*^*-/-*^ mutant cells are stronger supercompetitors than the simultaneous *puma*^*-/-*^*; noxa*^*-/-*^ cells, suggesting that other downstream targets are involved in *p53*^*-/-*^ cells competitive status, such as other BCL-2 proteins or MYC. Regarding the mechanism by which P53 and BCL2 proteins PUMA and NOXA regulate pluripotent cell competitive fitness, we have shown that PUMA is strongly localized to mitochondria. Interestingly, knocking out *p53* in *bmpr1*^*-/-*^ increased the membrane potential over the level of the *WT*, indicating that P53 acts downstream BMP deficiency to affect mitochondrial activity [21]. An interesting possibility then is that P53-PUMA-NOXA regulation of cell competitive fitness is mediated by the role of this pathway on mitochondrial activity.

The knowledge on the upstream regulation of the P53 pathway in pluripotent cells remains incomplete. Different types of stresses have been associated to CC, such as oxidative or proteotoxic stress in different models [47,49]. We have explored DNA damage and oxidative stress by measuring the presence of double strand breaks (DSBs) and using the molecular probe dihydroethidium (DHE) respectively, but we did not identify a correlation between these types of stress and MYC levels in ES cells (S9 Fig). Apart from stress, we have shown that P53-PUMA and MYC are regulated by the pluripotency status. Although we did not explore in detail whether P53-PUMA can exert a role in pluripotency, P53 has been previously associated with ESC differentiation [37,55–59]. We found that in the absence of *p53*, ESCs showed higher levels of p-ERK. This could be interpreted as a direct effect of P53 preventing ESC differentiation, thereby promoting the naive pluripotent state. Alternatively, given that in these culture conditions naive cells tend to kill primed cells by Cell Competition [6], a possible mechanism for the increase in p-ERK would be the inhibition of primed-cell death, which would lead to their accumulation. Against this idea, in the same test, elimination of *puma* or *noxa* does not change the p-ERK levels (Fig 6G), suggesting that these death pathways are not involved in the role of P53 on ES cell differentiation.

The widespread expression of PUMA in early mouse embryos and ESCs appears exclusively related to pluripotency and CC. *In vivo*, only epiblast cells show this widespread PUMA expression profile whereas its expression is shut down in gastrulating cells. This change in expression during gastrulation might be related to a shift in its function from a factor that regulates fitness to a cell death executioner. High expression of PUMA in a heterogeneous pattern therefore seems to be related to pluripotency *in vivo* and *in vitro*. In this respect, the recent mitochondrial metabolic role described for PUMA in cancer cells assimilates these to pluripotent cells of the early embryo [36].

Interestingly, we have reported that P53 exerts an inhibitory effect over MYC *in vitro* and therefore, P53 occupies a high position in the pluripotent cell competitive fitness pathway. Nonetheless, ES cells still exhibit a MYC heterogeneous expression pattern in the absence of P53, indicating that additional factors contribute to the expression and variability of MYC in ES cells but there should be other factors regulation MYC expression. While we also observed a negative correlation *in vivo* between the levels of P53/PUMA and MYC at E6.5, eliminating *p53* in embryos was not sufficient to increase MYC levels. Additional pathways should thus be acting *in vivo* to regulate MYC expression. The previously described regulation of MYC by TEAD-YAP in early mouse embryos and fibroblasts [22,60] suggests that the Hippo pathway is a strong candidate in this context. Notably, *puma*^-/-^ cells or double knockout *puma*^*-/-*^, *noxa*^*-/-*^ cells do not present changes in MYC expression, and MYC overexpression does not modulate P53 or PUMA expression, indicating that MYC and PUMA/NOXA regulate independent branches of P53-induced CC (S8 Fig). In the future, experiments combining gain and loss of function for each pathway will determine whether MYC and PUMA/NOXA act cooperatively on a similar component of competitive fitness or regulate completely independent aspects of this cellular feature (S8 Fig).

## Materials and methods

### Ethics statement

Animals were handled in accordance with Centro Nacional de Investigaciones Cardiovasculares (CNIC) Ethics Committee, Spanish laws and the EU Directive 2010/63/EU for the use of animals in research. All mouse experiments were approved by the CNIC and Universidad Autónoma de Madrid Committees for “Ética y Bienestar Animal” and the area of “Protección Animal” of the Community of Madrid with references PROEX 220/15 and PROEX 144.1/21.

### Cell line generation

The *Myc*^*GFP/GFP*^ allele was described in [61] and the mES cell line carrying the allele was described in [6]. The MYC overexpressing cell line *iMOS*^*T1-MYC*^ was described in [4]. *Myc*^*-/-*^ ES cell line was generated in our lab by recombining the *Myc*^*flox*^ allele. *P53*, *puma* and *noxa* knockout ES cell lines were generated with CRISPR-CAS9 technology using the *Myc*^*GFP/GFP*^ ES cell line. Two crRNA sequences were employed per gene to generate a deletion in the gene sequence. The web tool CRISPOR (http://crispor.tefor.net/crispor.py) [62] was used for the design of the crRNAs which are indicated in S1 Table. In the case of *p53*, the targeted region included the DNA binding domain, the nuclear localization sequence and the oligomerization domain. Regarding *puma*, the deleted region covers the majority of exons 1 and 2, including the BH3 domain and the Ser96 and Ser106 residues, recently described as key for PUMA-metabolic functions [36]. In the case of *noxa*, the removed region consisted in exons 2 and 3, including BH3-1 and 2 domains.

crRNAs and tracrRNA were purchased from IDT while CAS9 protein was expressed and purified by the Pluripotency Cell Unit at CNIC. To generate each knockout cell line, 1x10^6^ Myc^GFP/GFP^ cells were electroporated together with the ribonucleoprotein (RNP) complex, formed by the guide RNA (crRNA + tracrRNA) and the CAS9. Cells were electroporated with the Neon Transfection System (Thermo Fisher Scientific). A *tdtomato*-expressing plasmid was used as a reporter, so that 24h after the electroporation, tdTomato positive cells were sorted by FACS. tdTomato expressing cells displayed due to the electroporation, allowing the *tdtomato* plasmid to get into and being expressed, being more likely that the RNP complex entered into these cells. Then, individual cells were expanded into single colonies and knockouts clones were screened by PCR using the primers detailed in S1 Table.

### Animals

Wild-type mice were on a CD1 out-bred genetic background. *p53*^*-/-*^ mice were generated by crossing heterozygous *p53*^*tm1b/+*^ mice, previously described in (http://www.informatics.jax.org/allele/MGI:6120822) [63]. *p53*^*tm1b/+*^ animals were kindly provided by Ignacio Flores. *P53*^*tm1b*^ animals were genotyped by PCR using the primers included in S1 Table.

### Embryo retrieval

Midday of the day when vaginal plug was detected was considered gestational day 0.5 (E0.5). Females were culled by CO_2_ inhalation and the uterus was transferred to DMEM media #41965–039 (ThermoFisher) at 37°C. Embryo extraction at E3.5 was performed by flushing the blastocysts out of the uterus under a dissection scope using a 1ml syringe with a 23-G needle. Blastocysts were transferred to KSOM media MR-101 (Merck) with a mouth pipette. Tyrode´s solution #T1788 (Merck) was used during a few seconds to dissolved zona pellucida. Blastocysts were then washed in PBS 1% FBS and fixed in paraformaldehyde (PFA 2%) dissolved in PBS 1% FBS overnight at 4°C. Eventually, blastocysts were washed in PBS + Triton-X100 0,1% 1% FBS. For E5.5-E.7 embryos extraction, working under the scope and using precision forceps (Dumont #55 0.05x0.02mm) (FST), muscular uterine walls were carefully ripped. After that, both the decidual layer and the Reichert´s membrane were removed and embryos were fixed in paraformaldehyde (PFA) (Merck) 2% in PBS overnight at 4°C. After fixation, embryos were washed in PBS several times.

### Cell culture

Mouse embryonic fibroblasts (MEFs) were used as feeder cells for the ESCs. Fibroblasts were initially extracted from E13.5 CD1 embryos by the Pluripotent Cell Technology unit at CNIC. 5 million fibroblasts were then plated on a 100mm plate in MEFs medium (described below) and passaged to three 150mm-plate 48h later. After 3–4 days, MEFs were inactivated using mitomycin C #M4287 (Sigma) during 2.5h and washed 3 times using PBS. Finally, MEFs were trypsinized (Trypsin-EDTA 10x, Gibco) and frozen (1,2 million cells in 1ml of freezing medium). Upon inactivation, MEFs were plated on 0,1%-gelatin coated plates. For cell expansion, 1 million mouse embryonic stem cells (mESCs) were plated on a 35mm-plated previously covered by inactivated MEFs. After 48 hours, cells were passaged on a 100mm-plate covered by inactivated MEFs and cells were trypsinized and frozen two days later (1.2 million cells in 1ml volume per cryovial). To perform experiments, ESCs were thawed on a 35mm-plate covered by inactivated MEFs. After 2 days, MEFs depletion is performed and ESCs are transferred to a 0,1%-gelatin coated 35mm-plate. MEFs depletion is based in the different attachment of MEFs and ESCs, so after cells are trypsinized, MEFs attach in 1-2 hours, while ESCs can be transferred to a new plate. 0.7 million cells were cultured on a 35mm-plate and 0.18 million on a 12-wells plates. For freezing, cells were resuspended in MEFs medium and freezing medium was carefully added in a final proportion 1:1 and aliquot into cryovials. Cryovials are kept in a freezing container (Nalgene) at -80°C and transferred into liquid nitrogen after 24h.

### Medium composition

MEF medium contains high glucose DMEM (#41965, LifeTech), 15% Fetal Bovine Serum (FBS), 1% sodium pyruvate (#11360070, ThermoFisher), 1% Penicillin/Streptomycin (10,000U/ml; 100x), 0,1% 2-beta-mercaptoethanol (50mM). mESC medium (FBS+LIF) consisted of MEF medium plus 1% non-essential amino acids (100x) and LIF (leukemia inhibitor factor). LIF was removed to induce mESC differentiation. FBS was substituted by KnockOut serum replacement (here referred as SR) (#10828, Invitrogen) to get SR+LIF medium. Finally, the inhibitors CHIR99021 (#04-0004-02, Stemgent) and PD0325901 (#04–0006, Stemgent) were added at 3μM and 1μM, respectively, to the FBS+LIF medium to obtain “2i medium”.

### Competition assays

0.18 million cells were plated in co-culture or separated using 12-well plates and FBS medium without LIF. At every time point, cells were trypsinized and counted using a Neubauer chamber (Sigma-Aldrich). The percentage of fluorescent and non-fluorescent cells in co-culture was determined by flow cytometry.

### RT-PCR

For RNA extraction, 4 million cells per ml were resuspended in TRI Reagent (Invitrogen) for 5min at room temperature (RT). Afterward, ethanol was added in a 1:1 volume proportion and vortex. “Direct-zol RNA Miniprep kit” (R2051, Zymo Research) was used to extract the RNA according to the manufacturers. Finally, RNA was stored at -80°C. To perform the cDNA reverse transcription, we used 1μg of RNA and the “High Capacity cDNA Reverse Transcription” Kit (4368814, ThermoFisher). Eventually, we performed a qPCR using “Sybr Green” (#4472903, Invitrogen). The primers for the qPCR reaction were purchased from “KiCqStart SYBR Green Primers” (Sigma-Aldrich) and the gene *gadph* was chosen as a control.

### Whole-mount embryo immunofluorescence

E3.5 whole-mount immunostaining was performed using 4-well plates and a mouth pipette. Triton X-100 0,1% and FBS 1% was added to the PBS and the blocking solutions to avoid blastocysts getting attached to the plate. E5.0-E7.5 embryo immunostaining was performed using 35mm plates and/or round bottom 2ml Eppendorf tubes using a micro-pipette with end-cut tips to avoid excessive pressure when transferring the embryos. Both E3.5 and E5.0-E7.5 embryos were permeabilized using 0,5% PBT (PBS + Triton X-100 0,5%) for 20min at RT. Embryos were washed in PBT 0,1% and blocked using 10% goat serum (Gibco-BRL Life-Technologies) in 0,3% PBT during 1 hour at RT. Embryos were incubated with primary antibodies overnight using blocking solution at 4°C. Embryos were then washed several times with PBT 0,1% and incubated with the secondary antibodies, Wheat Germ Agglutinin (1:500) (ThermoFisher) for plasma membrane staining and/or DAPI (1:1000) using blocking solution for 1 hour at RT. Finally, embryos were washed several times and embedded in mounting media. To avoid the embryos to collapse due to the different density between 0,1% PBT solution and mounting media, the mounting media was diluted in serial dilutions using 0,1% PBT, (25, 50, 80 and 100% mounting media) and the embryos were transferred through the different dilutions. For E.5.0-E7.5 embryos, we used VectaShield mounting media (H-1000-10, Vector Laboratories) while, for E3.5, liquid Abberior (MM-2013, Abberior) mounting media was used.

### mESCs immunofluorescence

0.18–0.25 million ES cells were plated on 35mm-glass bottom dishes (MatTek), previously coated using human fibronectin (#354008, Corning) according to the manufacturers ON at RT. Two days after plating, cells were washed with PBS and fixed with PFA 2% ON at 4°C. For mESCs, a similar procedure than with embryos was carried out, but permeabilization was reduced to 10 minutes and Vectashield was used as mounting media. Primary and secondary antibodies were incubated in a 100μl volume. To prevent evaporation during the primary antibody overnight incubation, plates were stored inside a humidity chamber. For 594-conjugated cleaved-CASP3 (#8172, Cell Signalling) immunostaing, we proceed as described by the manufacturers (Protocol Id: 182) (https://www.cellsignal.com/products/antibody-conjugates/cleaved-caspase-3-asp175-d3e9-rabbit-mab-alexa-fluor-594-conjugate/8172). To induce activation of P53, ESCs were exposed to etoposide (Sigma) 40μM during 10 hours or Nutlin-3 (BioVision) 5–30μM during 12 hours previous to fixation. For immunostaining of ESCs in suspension, ES cells were trypsinized and fixed with PFA 2% in PBS during 1 hour at 4°C. Saponin substituted Triton-X as a permeabilizing agent and is added at 0,1% in all solutions. After the immunostaining, ESCs were washed and resuspended in 200μl of PBS and analyzed by flow cytometry. Primary antibodies are summarized in S2 Table.

### Caspase activity measurement

FLICA 660 Caspase-3/7 (BIORAD) was used to measure apoptosis according to manufacturers.

### Cell cycle

Propidium iodide was used to determine the cell cycle. 0.2 million cells were trypsinized and resuspended drop-by-drop with 500μl of ethanol 70% at -20°C and stored for 24h at -20°C. Subsequently, cells were washed in PBS twice and resuspended in 200μl of PBS containing propidium iodide at 50μg/ml. Cells were analyzed by flow cytometry. Dean- Jett-Fox model was used for this analysis. In those samples containing *tdtomato*-positive cells DRAQ5 (65 0880–96, Invitrogen) was used instead to determine the cell cycle according to manufacturers.

### Immunoblot

Cells were lysed with RIPA buffer containing (25x) protease inhibitor (Roche) for 30min at 4°C. Approximately 1.5 million cells were lysed at a concentration of 3 million cells per ml. Protein concentration was measured using BSA (Sigma-Aldrich) serial dilutions and the “DC Protein Assay” kit (BioRad). Absorbance was measured at 690nm using a microplate spectrophotometer. Proteins were separated via 12% SDS-PAGE under reducing conditions and transferred to a polyvinylidene difluoride (PVDF) membrane using “Wed blotting system” (BioRad). After incubation with primary and secondary antibodies, protein signal was detected via chemiluminescence using the “Pierce ECL Western Blotting Substrate” kit (Thermo-Fisher).

### Equipment

Confocal microscopy was performed using a Leica TCS SP8 coupled to a DMi8 inverted confocal microscope Navigator module equipped with white light laser. A HC PL Apo CS2 40x/1.3 oil objective and 1024x1024 pixels, A.U. set to 1 were commonly used. For Super-resolution microscopy, a Leica gated STED-3X- WLL SP8 and a HC PL APO CS2 100x/1.40 oil objective was used. Alexa Fluor 514 and Alexa Fluor 568 secondary antibodies were used for this technique. Flow Cytometry was performed using a BD LSRFortessa Special Order Research Product (laser wavelengths 405, 488, 561, 633). Additionally, ESCs were sorted using a BD FACSAria II and Synergy 4L cell sorter.

### Image analysis

Confocal images were analyzed using FIJI [64] (https://imagej.net/Fiji). For nuclear segmentation, we took advantage of DAPI/TO-PRO-3 staining. Nuclei masks were created applying the “default Threshold tool” and manually corrected to ensure that segmented objects correspond to individual cells and discard mis-located, apoptotic or mitotic cells. Finally, “Analyzed particle” tool was used to identify the regions of interest (ROIs).

For cytoplasmic segmentation, we used the WGA membrane staining. We applied a “Gaussian Blur” filter (scaled units 2) and the “Find Maxima” tool (configuration: output type, segmented particles; light background) to create a mask. Afterwards, manual correction was performed to ensure that segmented objects correspond to individual cells, and we applied “Analyzed particles” to identify the ROIs. Finally, we subtracted the nuclear area (obtained as described above) to the whole cell region. To couple the cellular and nuclear ROIs from the same cell, a macro was designed together with the CNIC Microscopy Unit (S1 Data).

To quantify nuclear foci, ROIs corresponding to a nucleus were selected and processed using the “Find maxima” tool (output = count). This process was automated by running a macro with a loop (S1 Data).

To study PUMA and MYC correlation in E6.5 mouse embryos, MYC levels were normalized per Z slice and embryo to avoid depth-dependent loss of signal and variation among different embryos. Statistical analysis was performed using linear mixed models using lme4 R library, p value = 6.81x10^-10^. This type of analysis was also performed for the P53-PUMA correlation at E3.5, p = 0.105 and P53-MYC correlation at E3.5. Embryo was set as random variable and either P53, PUMA or MYC-classification and Z-position as covariates to simultaneously adjust for the two factors. Coefficients represent either the quantitative increase in the response variable (log2(MYC)) per unit increase in the independent variables (either log2(P53) or PUMA-classification variables) and their associated p-values show the significance of such coefficients under the null hypothesis of them being 0.

### Statistical analysis

Parametrical T student test was performed to compare two groups of data. For comparisons with more than two groups of data, ordinary one-way ANOVA with Šídák’s multiple comparisons test was used. When several groups were compared along time, two-way ANOVA with Šídák’s multiple comparisons test was used. One-sample test (Wilcoxon test) was used to compare a group of data with a hypothetical mean. Comparison and graphs were made with Graph Pad Prism 8.4.3 statistical analysis software. Adjusted values of P<0,05 were considered statistically significant.

### RNAseq meta-analysis

RNAseq data for meta-analysis were initially described in [6]. For the enrichment analysis, we used the web server gProfiler (version e108_eg55_p17_9f356ae) with g:SCS multiple testing correction method applying significance threshold of 0.05 [65]. For the analysis, we previously selected those genes upregulated in MYC-low cells and we filtered those genes with more than 3 reads after normalization. For the selection of candidate genes from our RNAseq data related to the P53 pathway and associated to apoptosis/cell stress, we took advantage of Gene Ontology and GO Annotation, using the QuickGO (https://www.ebi.ac.uk/QuickGO/) developed by the EMBL’s European Bioinformatics Institute (EMBL-EBI) (Tables C and D in S3 Table).

GO terms related to P53 pathway:

GO:0002039 p53 binding

GO:0072331 signal transduction by p53 class mediator

GO:1901796 regulation of signal transduction by p53 class mediator

GO:0072332 intrinsic apoptotic signaling pathway by p53 class mediator, regulation of DNA damage response, signal transduction by p53

GO:0043516 regulation of DNA damage response, signal transduction by p53 class mediator

GO:0002039 p53 binding

GO terms related to apoptosis:

GO:0042981 regulation of apoptotic process

GO:1900119 positive regulation of execution phase of apoptosis

GO:1900118 negative regulation of execution phase of apoptosis

GO:0097190 apoptotic signaling pathway

GO:0097194 execution phase of apoptosis

For the Volcano plot representation, we used the web app VolcaNoseR tool [66] (https://huygens.science.uva.nl/VolcaNoseR2/) maintained by Joachim Goedhart and Martijn Luijsterburg. We filtered the genes with more than 3 reads and discard those without a term in the MGI (Mouse Genome Informatics) database and those with an annotation starting by Gm or ending in Rik. The genes are represented by the log_2_ Fold change and the -log_10_ AdjPValue.

## Supporting information

S1 Fig Pluripotency stages *in vivo* and *in vitro*. **A**. In mice, pluripotency starts before implantation, at the blastocyst stage. Inside the embryo, a population of cells (depicted in blue) known as the inner cell mass (ICM) embodies pluripotency. Pluripotency is not a single status but a sequence of states. Shortly before implantation, the ICM segregates into the epiblast and the primitive endoderm. At this stage epiblast cells acquire the so-called "naive" pluripotency characterized by an open chromatin and lack of lineage commitment epigenetic or transcriptional markers. After implantation, the epiblast evolves through several days of “formative pluripotency”, during which, cells prepare for differentiation. Around E6.5, cells progress into a "primed" state in which they are ready for differentiation. The onset of gastrulation definitely brings primed cells into the differentiation program towards the three primordial germ layers. These diverse pluripotent states can be recreated in vitro. ESCs are derived from the preimplantation ICM or naive epiblast. When cultured in 2i medium, they can expand maintaining naive pluripotency. Using SR+LIF or FBS+LIF, ESC cultures exhibit a more diverse status, with mixed populations showing different degrees of evolution from the naive to the primed status. By using FBS instead of SR cells are allowed to evolve further into formative pluripotency. LIF removal from these conditions, leads to transient acquisition of the primed status followed by differentiation. Epiblast Stem Cells (EpiSCs) can be derived from E5.5 embryos, and can be maintained in vitro in a "primed" status by Activin-A and FGF.
(TIF)

S2 Fig Pathway enrichment analysis and identification of candidate genes downstream P53. **A.** Dot plot and table showing the most enriched pathways and terms associated with our RNAseq data. **B.** Confocal images showing P53 expression in normal conditions and after the exposure to etoposide. **C.** Volcano plots showing the genes from our RNAseq data. Different candidate genes related to the P53 pathway and apoptosis were highlighted. **D.** Bar graph showing the MYC-low versus MYC-high ratio from a qPCR of the indicated candidate genes. Error bars show standard deviation.
(TIF)

S3 Fig BCL-2 family pro-apoptotic protein PUMA displays a higher expression in MYC-low cells. **A.** Schematic representation of the different BCL-2 protein subfamilies and mechanism of action (**B**). **C.** (left) Western blot of PUMA expression in MYC-high and MYC-low population and quantification (right). **D.** RNAseq data analyses indicating the normalized expected counts of the 4 isoforms of *puma*.
(TIF)

S4 Fig MYC does not regulate P53 or PUMA. **A.** Confocal images showing P53 and PUMA expression with or without etoposide and quantification (**B**). **C.** Confocal captures showing MYC levels in *WT* and *myc*^*-/-*^ cells (left) and quantification of P53 and PUMA levels in *WT* and *myc*^*-/-*^ cells (right). Each dot represents one *WT* or *myc*^*-/-*^ clone. At least 377 cells were analyzed for each ES cell line. **D.** Bar graph showing an independent experiment similar to that in C, analyzed by flow cytometry. **E.** Schematic representation of the iMOS^MYC^ system [4] (left). Confocal images showing P53 and EYFP expression and quantification of P53 and PUMA levels in *WT* cells and cells overexpressing MYC (right).
(TIF)

S5 Fig P53 activation using Nutlin3 induces PUMA upregulation and MYC inhibition. **A.** Confocal images showing P53, PUMA and MYC levels in normal conditions and after Nutlin3 treatment and quantification (**B**). **C.** MYC levels upon treatment with different doses of Nutlin3, analyzed by flow cytometry.
(TIF)

S6 Fig P53, PUMA and MYC expression in the early mouse embryo. **A.** MYC expression in *WT* and *p53*^*-/-*^ E6.5 mouse embryos (left) and quantification (right). Each bar represents one epiblast. The two graphs represent two independent experiments. **B.** Confocal captures showing P53 and PUMA expression in E3.5 mouse embryos and quantification (**C**). Sixteen blastocysts were used for this quantification. **D**, P53 and MYC expression in E3.5 embryos and quantification (**E**). This includes two independent experiments represented in colors: dark blue; 3 embryos, and yellow; 5 embryos. **F**, P53 and MYC expression in *WT* and *p53*^*-/-*^ E3.5 mouse embryos (left) and quantification (right). Each bar represents one embryo and dots represent individual cell quantification. White arrow indicates a *p53*^*-/-*^ embryo.
(TIF)

S7 Fig P53, PUMA and NOXA have a role in apoptosis but not in ESC proliferation. **A.** Percentage of active CASP3/7 using the fluorogenic CASP substrate FLICA. **B.** Contour dot plot showing phH3 positive and negative cells populations (left). Bar graph showing percentage of positive phH3 cells in the indicated ES cell lines. Each dot represents one different clone.
(TIF)

S8 Fig Model for integration of the P53 and Myc pathways in pluripotent Cell Competition. **A.** Stress, pluripotency status or anabolic capacity have been described as important elements in Pluripotent Cell Competition [4,6,21]. P53 is a well described component in Cell Competition in different models, including pluripotent cells [23,41] and a sensor of cellular stress. Here, we have identified several candidate genes downstream P53 that form part of competitive fitness and may involve different mechanisms, like mitochondrial function, autophagy or Ca^2+^ homeostasis. **B.** The absence of P53, PUMA or the simultaneous deletion of PUMA and NOXA is enough to trigger competitive interactions and outcompete WT cells.
(TIF)

S9 Fig DNA damage and oxidative stress do not correlate with MYC levels. **A.** MYC expression and P53BP1 foci in ES cells and quantification (**B**). **C.** DHE and MYC expression in ESCs and quantification (**D**).
(TIF)

S1 Table Sequences of the oligonucleotides used in this study. (DOCX)

S2 Table Antibodies used in this study with their dilutions. (DOCX)

S3 Table Table A. RNAseq. List of genes with RNAseq results according to their Myc expression levels. Table B. RNAseq (filtered). Curated list of genes using the RNA sequencing data. This includes genes exhibiting negative fold changes (indicating overexpression in the MYC-low condition), a minimum of three reads and an adjusted p-value below 0.05. Table C. GO- Apoptosis & P53. List of genes linked to Gene Ontology terms pertinent to apoptosis and the P53 pathway. Table D. RNAseq candidates (GO filtered). List of genes comprising those genes from the curated RNAseq list in Table B, which are also associated with a GO term pertinent to apoptosis and the P53 pathway.
(XLSX)

S1 Data FIJI macro for the segmentation of the cytoplasm. (DOCX)
