# Supplementary figures and images for: P53 and BCL-2 family proteins PUMA and NOXA define competitive fitness in pluripotent cell competition
Source: PLoS Genet. 2024 Mar 15;20(3):e1011193. doi: 10.1371/journal.pgen.1011193 (PMC10971546; doi:10.1371/journal.pgen.1011193)

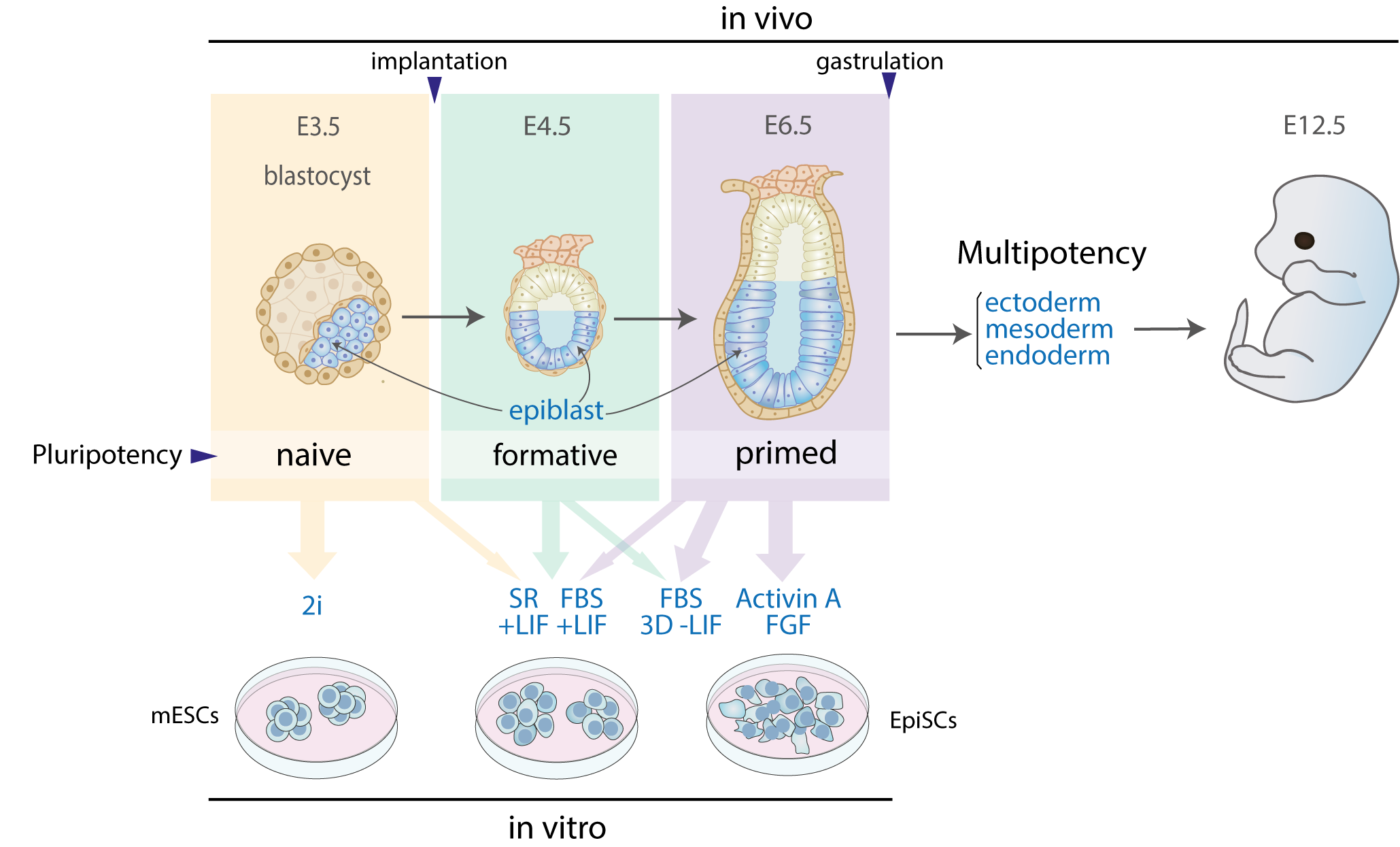

Supplement: S1 Fig — A. In mice, pluripotency starts before implantation, at the blastocyst stage. Inside the embryo, a population of cells (depicted in blue) known as the inner cell mass (ICM) embodies pluripotency. Pluripotency is not a single status but a sequence of states. Shortly before implantation, the ICM segregates into the epiblast and the primitive endoderm. At this stage epiblast cells acquire the so-called "naive" pluripotency characterized by an open chromatin and lack of lineage commitment epigenetic or transcriptional markers. After implantation, the epiblast evolves through several days of “formative pluripotency”, during which, cells prepare for differentiation. Around E6.5, cells progress into a "primed" state in which they are ready for differentiation. The onset of gastrulation definitely brings primed cells into the differentiation program towards the three primordial germ layers. These diverse pluripotent states can be recreated in vitro. ESCs are derived from the preimplantation ICM or naive epiblast. When cultured in 2i medium, they can expand maintaining naive pluripotency. Using SR+LIF or FBS+LIF, ESC cultures exhibit a more diverse status, with mixed populations showing different degrees of evolution from the naive to the primed status. By using FBS instead of SR cells are allowed to evolve further into formative pluripotency. LIF removal from these conditions, leads to transient acquisition of the primed status followed by differentiation. Epiblast Stem Cells (EpiSCs) can be derived from E5.5 embryos, and can be maintained in vitro in a "primed" status by Activin-A and FGF. (TIF) [file pgen.1011193.s001.tif]

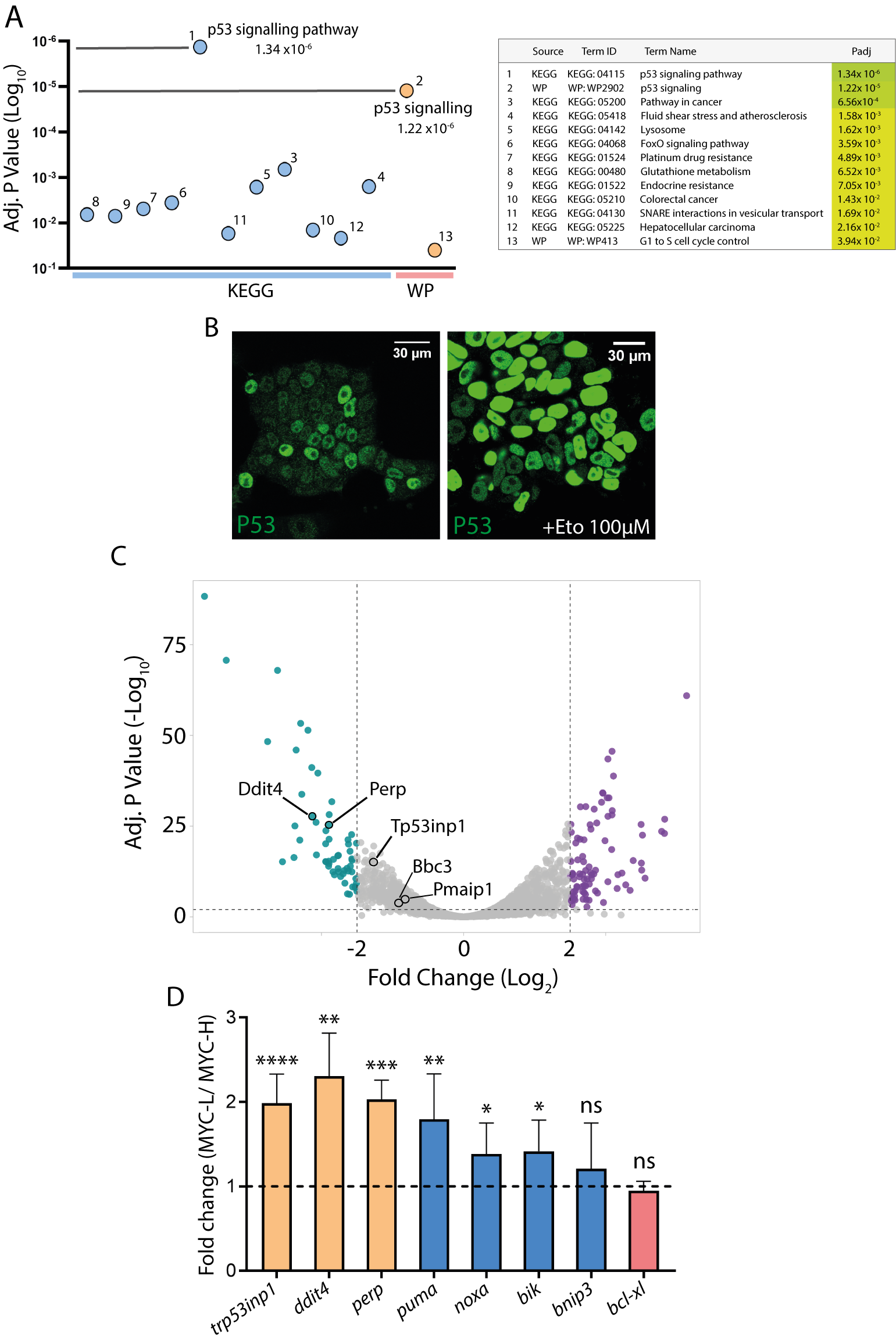

Supplement: S2 Fig — A. Dot plot and table showing the most enriched pathways and terms associated with our RNAseq data. B. Confocal images showing P53 expression in normal conditions and after the exposure to etoposide. C. Volcano plots showing the genes from our RNAseq data. Different candidate genes related to the P53 pathway and apoptosis were highlighted. D. Bar graph showing the MYC-low versus MYC-high ratio from a qPCR of the indicated candidate genes. Error bars show standard deviation. (TIF) [file pgen.1011193.s002.tif]

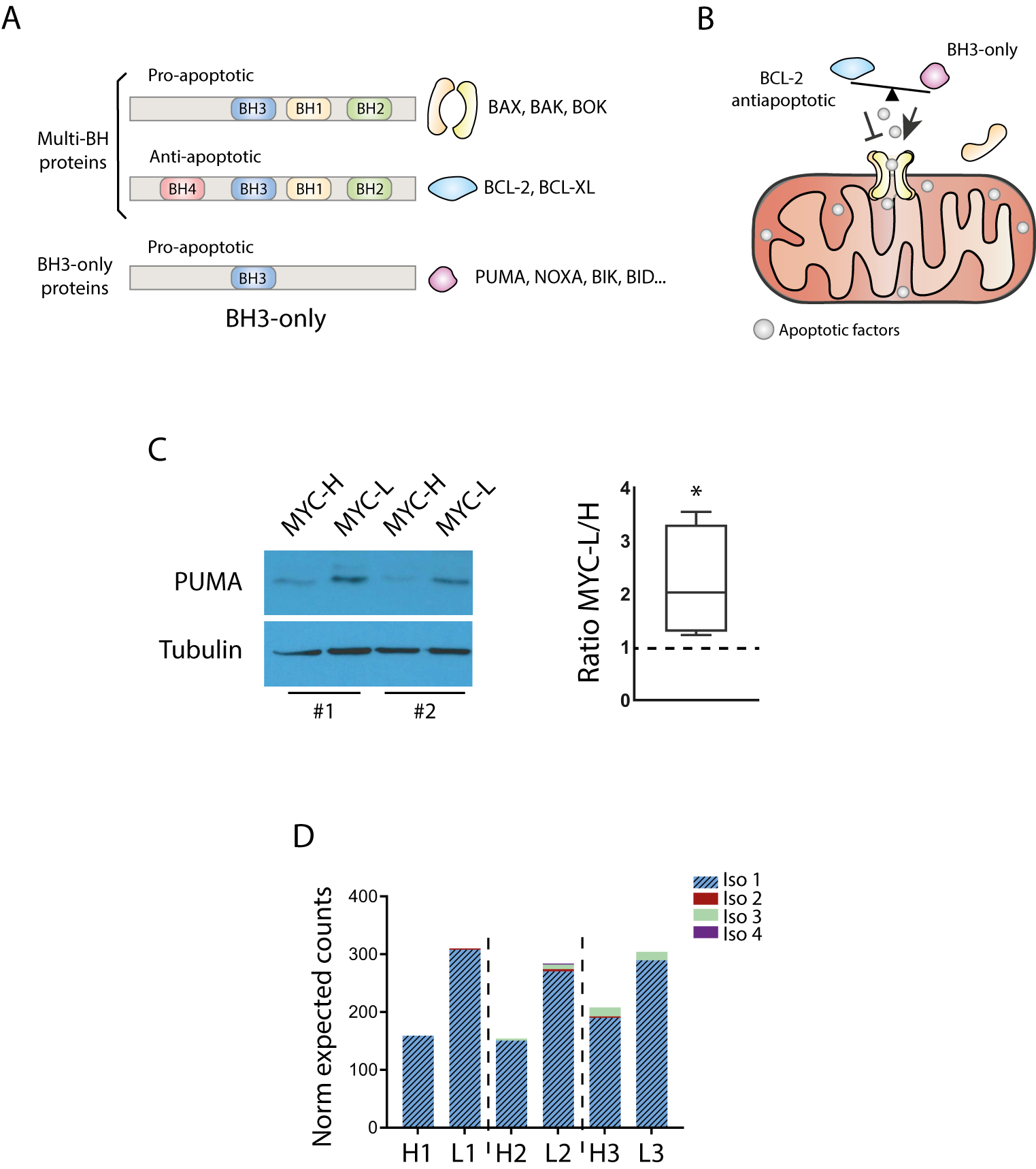

Supplement: S3 Fig — A. Schematic representation of the different BCL-2 protein subfamilies and mechanism of action (B). C. (left) Western blot of PUMA expression in MYC-high and MYC-low population and quantification (right). D. RNAseq data analyses indicating the normalized expected counts of the 4 isoforms of puma. (TIF) [file pgen.1011193.s003.tif]

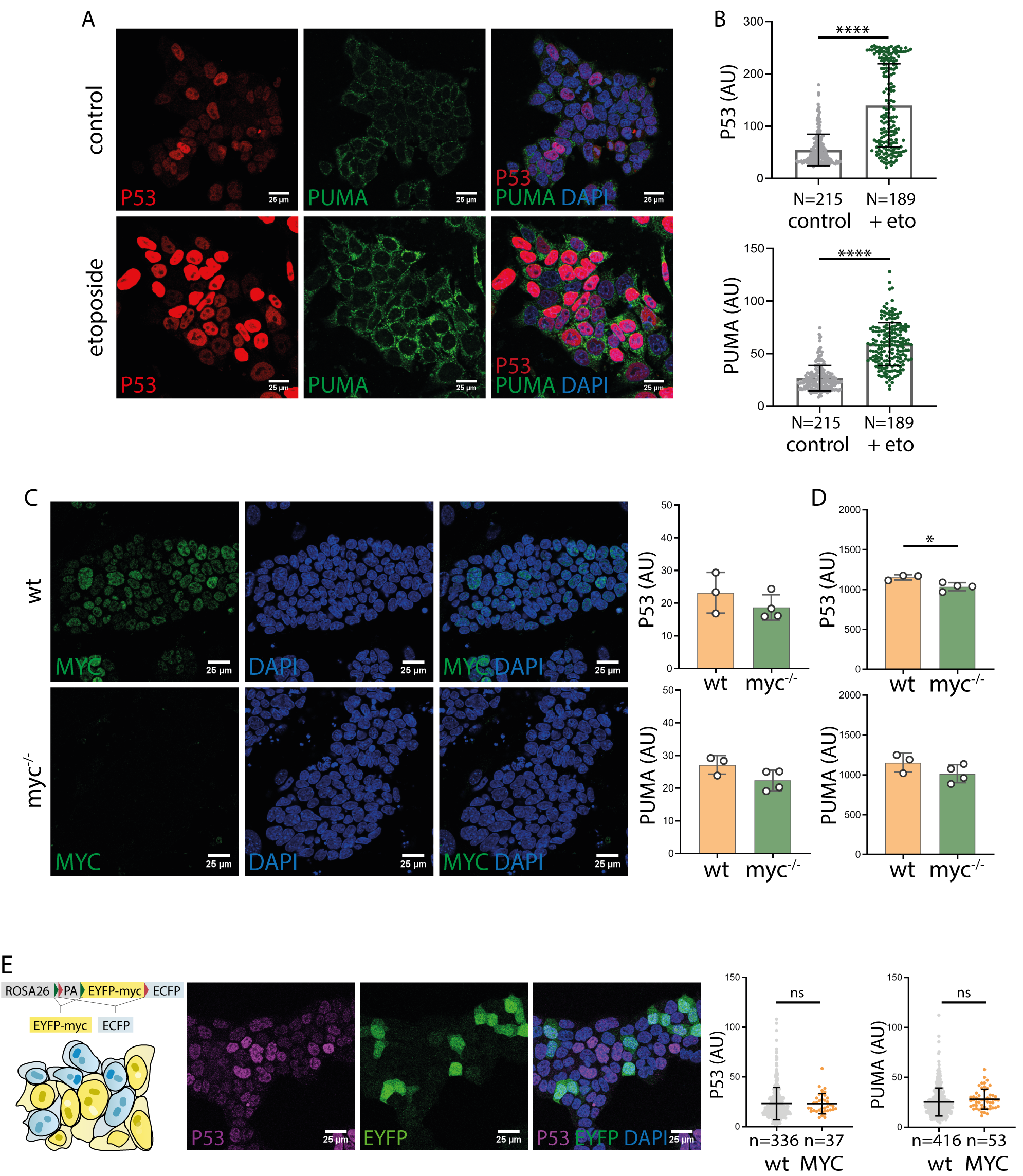

Supplement: S4 Fig — A. Confocal images showing P53 and PUMA expression with or without etoposide and quantification (B). C. Confocal captures showing MYC levels in WT and myc-/- cells (left) and quantification of P53 and PUMA levels in WT and myc-/- cells (right). Each dot represents one WT or myc-/- clone. At least 377 cells were analyzed for each ES cell line. D. Bar graph showing an independent experiment similar to that in C, analyzed by flow cytometry. E. Schematic representation of the iMOSMYC system [4] (left). Confocal images showing P53 and EYFP expression and quantification of P53 and PUMA levels in WT cells and cells overexpressing MYC (right). (TIF) [file pgen.1011193.s004.tif]

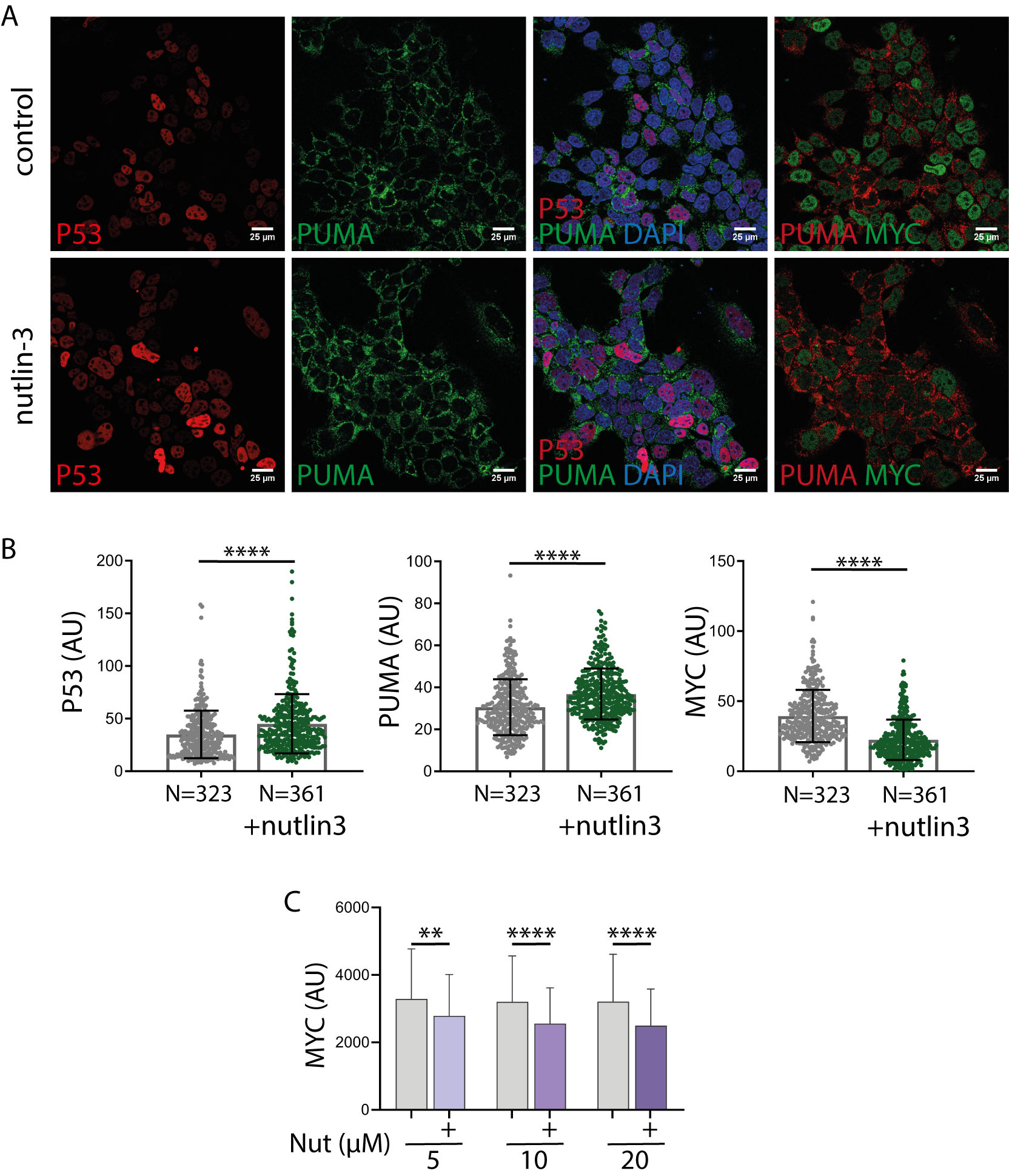

Supplement: S5 Fig — A. Confocal images showing P53, PUMA and MYC levels in normal conditions and after Nutlin3 treatment and quantification (B). C. MYC levels upon treatment with different doses of Nutlin3, analyzed by flow cytometry. (TIF) [file pgen.1011193.s005.tif]

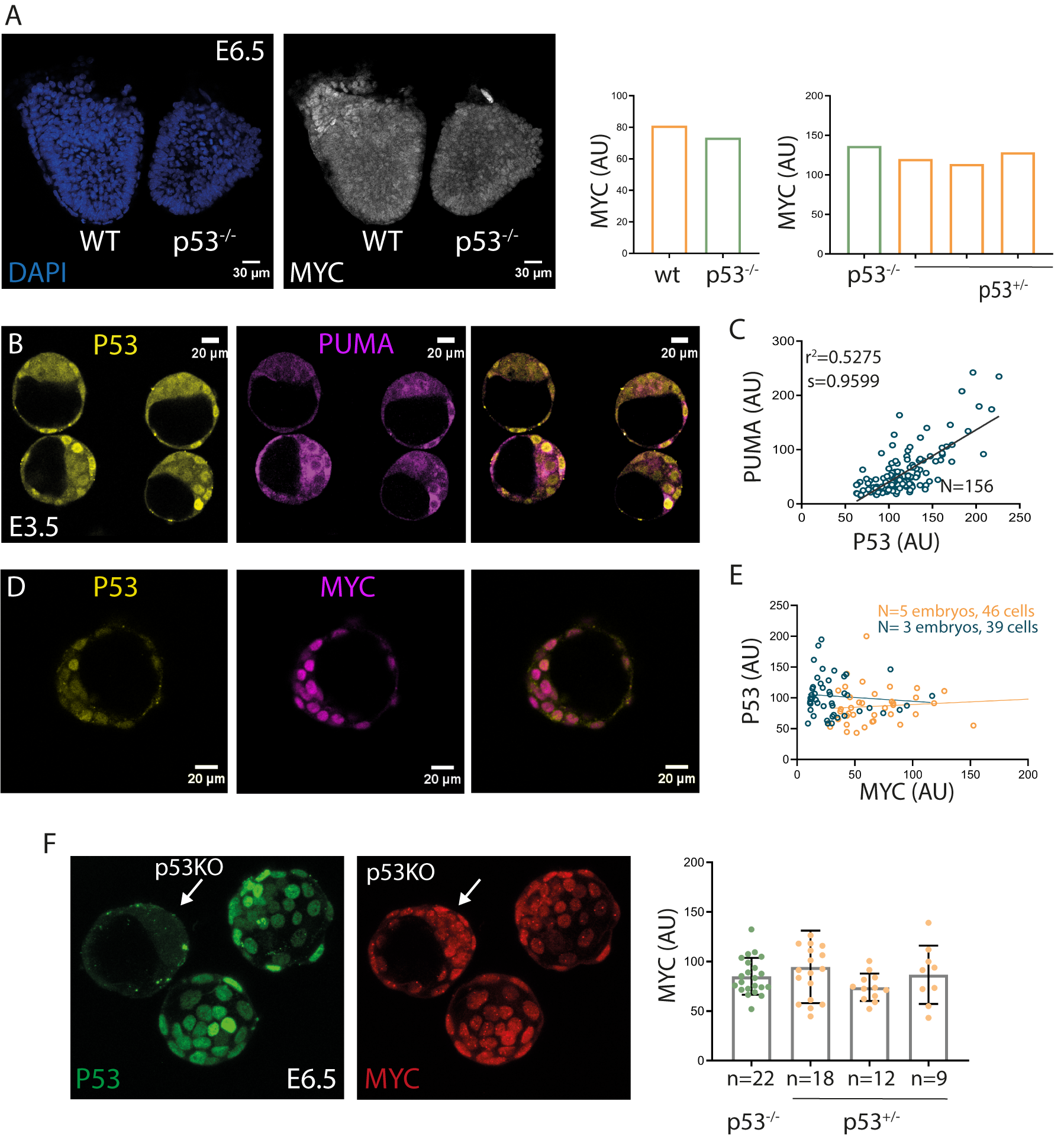

Supplement: S6 Fig — A. MYC expression in WT and p53-/- E6.5 mouse embryos (left) and quantification (right). Each bar represents one epiblast. The two graphs represent two independent experiments. B. Confocal captures showing P53 and PUMA expression in E3.5 mouse embryos and quantification (C). Sixteen blastocysts were used for this quantification. D, P53 and MYC expression in E3.5 embryos and quantification (E). This includes two independent experiments represented in colors: dark blue; 3 embryos, and yellow; 5 embryos. F, P53 and MYC expression in WT and p53-/- E3.5 mouse embryos (left) and quantification (right). Each bar represents one embryo and dots represent individual cell quantification. White arrow indicates a p53-/- embryo. (TIF) [file pgen.1011193.s006.tif]

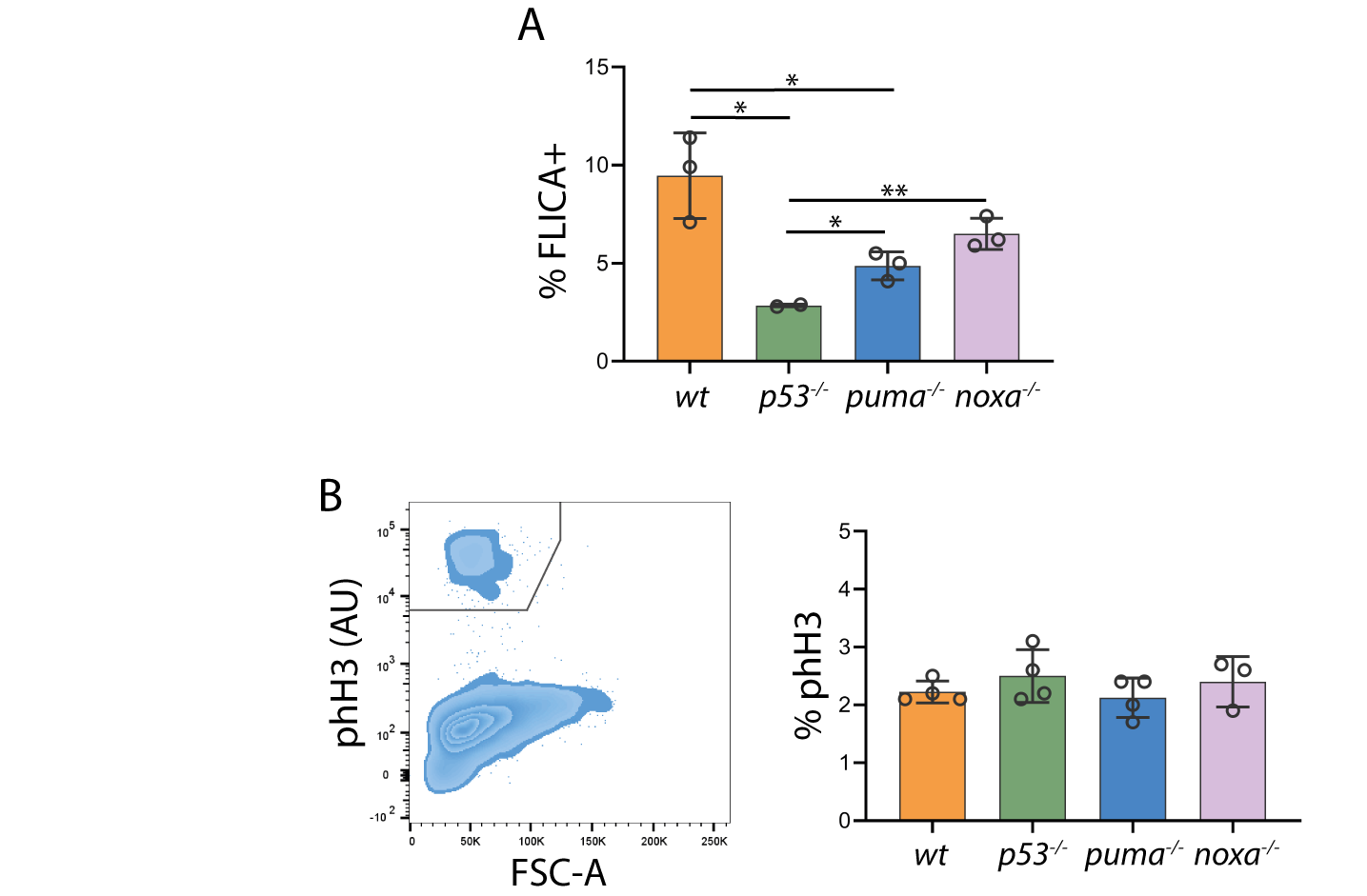

Supplement: S7 Fig — A. Percentage of active CASP3/7 using the fluorogenic CASP substrate FLICA. B. Contour dot plot showing phH3 positive and negative cells populations (left). Bar graph showing percentage of positive phH3 cells in the indicated ES cell lines. Each dot represents one different clone. (TIF) [file pgen.1011193.s007.tif]

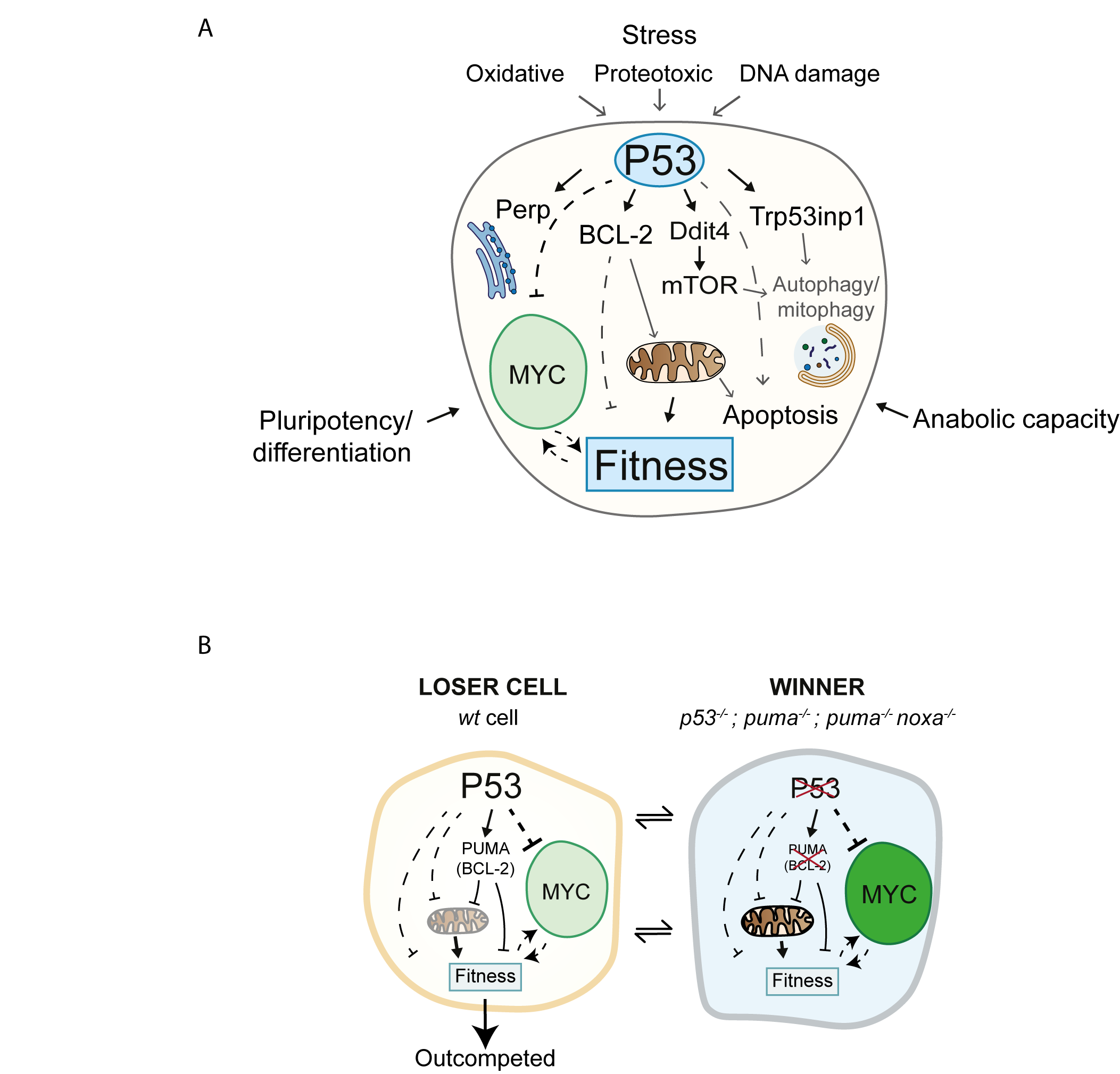

Supplement: S8 Fig — A. Stress, pluripotency status or anabolic capacity have been described as important elements in Pluripotent Cell Competition [4,6,21]. P53 is a well described component in Cell Competition in different models, including pluripotent cells [23,41] and a sensor of cellular stress. Here, we have identified several candidate genes downstream P53 that form part of competitive fitness and may involve different mechanisms, like mitochondrial function, autophagy or Ca2+ homeostasis. B. The absence of P53, PUMA or the simultaneous deletion of PUMA and NOXA is enough to trigger competitive interactions and outcompete WT cells. (TIF) [file pgen.1011193.s008.tif]

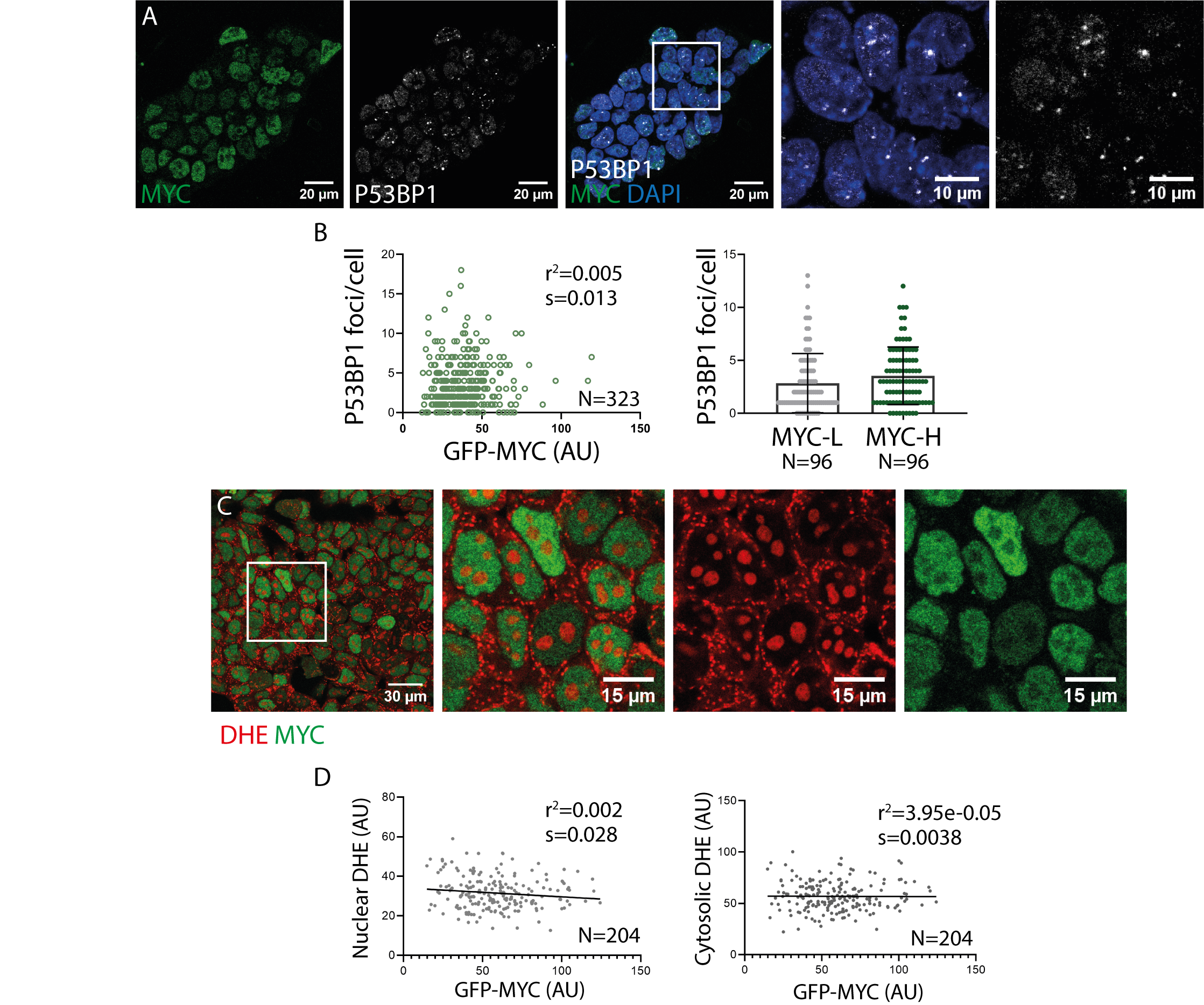

Supplement: S9 Fig — A. MYC expression and P53BP1 foci in ES cells and quantification (B). C. DHE and MYC expression in ESCs and quantification (D). (TIF) [file pgen.1011193.s009.tif]
