# Supplementary material for: P53 and BCL-2 family proteins PUMA and NOXA define competitive fitness in pluripotent cell competition
Source: PLoS Genet. 2024 Mar 15;20(3):e1011193. doi: 10.1371/journal.pgen.1011193 (PMC10971546; doi:10.1371/journal.pgen.1011193)
Supplement: S1 Table — (DOCX) [file pgen.1011193.s010.docx]

**TABLE 1. crRNA and primer sequences**

| **OLIGONUCLEOTIDE** | **FORWARD**  **/REVERSE** | **SEQUENCE (5’-3’)** |  |
| --- | --- | --- | --- |
| **Oligomers for crRNA** | | | |
| crP53 #1 |  | GGACAAGCCGAGTAACGATC AGG |  |
| crP53 #2 |  | TCTCGAAGCGTTTACGCCCG CGG |  |
| crPUMA #1 |  | TCGCGGGCTAGACCCTCTAC GGG |  |
| crPUMA #2 |  | CAACGCGCAGTACGAGCGGC AGG |  |
| crNOXA #1 |  | GGATGTGCTAATTTGCGAGT AGG |  |
| crNOXA #2 |  | AAGGAAGTTCCGCCGGTTGA TGG |  |
| **Primers for KO screening** | | | |
| P53 #1 | Forward | TTCCCACCCTCGCATAAGTTT |  |
| P53 #1 | Reverse | GAGGTCTGGGTAGAGCACCA |  |
| P53 #2 | Forward | AGGGGACGTGGAACTCTCTT |  |
| P53 #2 | Reverse | GCAGCCCTAAGCATCTAGCA |  |
| PUMA #2 | Forward | TTTGCTACAAACCCCAGACG |  |
| PUMA #2 | Reverse | GCATCCAGCAGATCCATTCCTT |  |
| PUMA #5 | Forward | CCTGGTGGGTTTTGCTACAA |  |
| PUMA #5 | Reverse | TAGCCCGGGATATAGGAGCC |  |
| NOXA #1 | Forward | AGGAGGGCATAAATGGGCAA |  |
| NOXA #1 | Reverse | ACTTCCCTAGCTCCACGACT |  |
| NOXA #3 | Forward | GAGGGGTACCAGAACAACCA |  |
| NOXA #3 | Reverse | CAAACGACTGCCCCCATACA |  |
| **Primers for Gibson assembly** | | | |
| Grx1-roGFP2 | Forward | TTCTTCCATTTCAGGTGTCGTGAGGAATTG- GATCCCCGGGATGGCCTCCACTCGTGTC |  |
| Grx1-roGFP2 | Reverse | ACAAATTTTGTAATCCAGAGGTTGATTGTCGAC- GAATTCGTTACTTGTACAGCTCGTCCATG |  |
| **Primers for *p53-/-* mice** | | | |
| p53f_12B7 | Forward 1 | TGGTTTGTGCGTCTTAGAGACAGT |  |
| pPNTf_2B5 | Forward 2 | CCAGCTCATTCCTCCCACTCA |  |
| p53r_1B3 | Reverse | AAGGATAGGTCGGCGGTTCAT |  |
