# Supplementary material for: P53 and BCL-2 family proteins PUMA and NOXA define competitive fitness in pluripotent cell competition
Source: PLoS Genet. 2024 Mar 15;20(3):e1011193. doi: 10.1371/journal.pgen.1011193 (PMC10971546; doi:10.1371/journal.pgen.1011193)
Supplement: S2 Table — (DOCX) [file pgen.1011193.s011.docx]

**Table 2. Antibodies**

| **ANTIBODY** | **HOST** | **DILUTION** | **BRAND** | **REFERENCE** |
| --- | --- | --- | --- | --- |
| Alpha tubulin (TU-02) | mouse | 1:1000 | Santa Cruz | sc-8035 |
| Cleaved Caspase-3 (Asp175) | rabbit | 1:100 | Cell Signaling | 9661 |
| Cleaved Caspase-3 (Asp175) (D3E9) (Conjugate- Alexa Fluor® 594) | rabbit | 1:75 | Cell Signaling | 8172 |
| GFP | chicken | 1:500 | Aves lab | AB_2307313 |
| GLUT1 | rabbit | 1:300 | Merk | 07-1401 |
| MYC | rabbit | 1:300 | Merk | 06-340 |
| NOX4 | rabbit | 1:150 | Ajay Shah’ lab |  |
| NOXA | mouse | 1:50 | Novus | NB600-1159 |
| NOXA | mouse | 1:50 | Santa Cruz | sc-56169 |
| P53 (1C12) | mouse | 1:500 | Cell Signaling | 2524 |
| Phospho-Histone H2A.X | mouse | 1:500 | Merk | 05-636 |
| Phospho-Histone H3 (Ser10) | mouse | 1:500 | Cell Signaling | 9706 |
| Phospho-p44/42 MAPK (Erk1/2) (Thr202/Tyr204) (D13.14.4E) | rabbit | 1:200 | Cell Signaling | 4370 |
| Phospho-S6 (Ser240/244) (D68F8) | rabbit | 1:800 | Cell Signaling | 5364S |
| PUMA (D7L9L) | rabbit | 1:400 | Cell Signaling | 24633 |
| TOM20 | rabbit | 1:500 | Santa Cruz |  |
| GFP | rabbit | 1:200 | Origene | R1091P |
| Phospho-p53 (Ser15) | rabbit | 1:100 | Cell Signaling | 9248 |
|  |  |  |  |  |
