# Supplementary material for: P53 and BCL-2 family proteins PUMA and NOXA define competitive fitness in pluripotent cell competition
Source: PLoS Genet. 2024 Mar 15;20(3):e1011193. doi: 10.1371/journal.pgen.1011193 (PMC10971546; doi:10.1371/journal.pgen.1011193)
Supplement: S1 Data — (DOCX) [file pgen.1011193.s013.docx]

**ANNEX I**

**1. CYTOPLASM MACRO**

n = getNumber(“How many nuclei”, );

match = newArray(n);

for(i=0; i < n;i++){

for(j=n; j< roiManager(“count”);j++){

roiManager(“Select”, j);

getSelectionBounds(x, y, width, height);

xc = x + width/2;

yc = y + height/2;

roiManager(“Select”, i);

roiManager(“Set Line Width”, 0);

roiManager(“Rename”, “Cell_” + i+1);

roiManager(“Select”, i);

if(Roi.contains(xc, yc)){

match[i] = j;

roiManager(“Select”, j);

roiManager(“Set Line Width”, 0);

roiManager(“Rename”, “Nuclei_” + i+1);

j = roiManager(“Count”); } }

roiManager(“Select”, newArray(i, match[i]));

roiManager(“XOR”);

roiManager(“Add”);

roiManager(“Select”, roiManager(“Count”)-1);

roiManager(“Set Line Width”, 0);

roiManager(“Rename”, “cytosol_” + i+1); }

**2. FOCI NUMBER MACRO**

run(“Duplicate...”, “duplicate channels=1”);

recuento=roiManager(“Count”);

punctae=newArray(recuento);

for(roi=0;roi<recuento;roi++){

roiManager(“Select”,roi);

roiManager(“Rename”,”N”+roi+1);

run(“Find Maxima...”, “noise=30 output=Count”);

punctae[roi]=getResult(“Count”,roi); }

//Crear tabla

run(“Clear Results”);

for(roi=0;roi<recuento;roi++){

setResult(“Puntae”,roi, punctae[roi]);

updateResults; }
